# A multimodal deep learning model for predicting impending rupture in symptomatic abdominal aortic aneurysms using CTA and clinical data

**DOI:** 10.3389/fcvm.2026.1771669

**Published:** 2026-04-07

**Authors:** Jiaxin Cheng, Sihan Wang, Zhiqiang Zhang, Ruoxi Gu, Xuan Wu, Yasong Wang, Yu Sun, Nan Wang, Xiaozeng Wang, Haibo Yu

**Affiliations:** 1 College of Medicine and Biological Information Engineering, Northeastern University, Shenyang, Liaoning, China; 2 National Key Laboratory of Frigid Zone Cardiovascular Disease, Cardiovascular Research Institute and Department of Cardiology, General Hospital of Northern Theater Command, Shenyang, Liaoning, China; 3 State Key Laboratory of Robotics, Shenyang Institute of Automation, Chinese Academy of Sciences, Shenyang, Liaoning, China; 4 Department of Radiology, General Hospital of Northern Theater Command, Shenyang, Liaoning, China

**Keywords:** abdominal aortic aneurysms, attention mechanism, explainable artificial intelligence, intervention timing, multimodal fusion

## Abstract

**Objective:**

In hemodynamically stable patients with symptomatic abdominal aortic aneurysms (AAA), timely diagnosis of impending rupture remains a critical challenge. To address this, we developed and validated an interpretable multimodal deep learning model to assess rupture risk and support emergency decision-making.

**Methods:**

This retrospective cohort study included 263 symptomatic AAA patients, with the most recent year's cases (*n* = 33) as an independent temporal test set. In the 230-patient development cohort, 75 impending rupture cases were matched 1:1 with 75 stable controls using propensity score for age, sex, and maximum aortic diameter. We developed a multimodal deep learning model that combines sequential CTA slices with six key clinical biomarkers through a bidirectional cross-attention (BCA) mechanism built on a ResNet-50 image encoder. For interpretability, we used Gradient-weighted Class Activation Mapping (Grad-CAM) and conducted pre-specified sensitivity analyses assessing robustness against endpoint decision-dependence, treatment-related data leakage, and domain shifts.

**Results:**

In the matched development test set (*n* = 30), our multimodal model achieved an area under the curve (AUC) of 0.898 with sensitivity and negative predictive value (NPV) both at 93.3%, offering a high safety margin for ruling out rupture. It markedly outperformed two pragmatic clinical baselines (clinical-rule model AUC: 0.751; CTA-sign model 0.778). This strong performance persisted in the independent temporal validation cohort (*n* = 33), where it attained an AUC of 0.880, sensitivity of 92.9%, and NPV of 87.5%. The proposed BCA fusion outperformed alternative architectures, and Grad-CAM visualizations were anatomically plausible in 78.8% of cases, supporting model interpretability.

**Conclusion:**

We developed and temporally validated an interpretable multimodal model that integrates CTA and clinical biomarkers to enable rapid AAA rupture risk stratification, offering a clinically relevant improvement in the safety and efficiency of emergency triage over current practice, pending prospective validation.

## Introduction

Rupture of an abdominal aortic aneurysm (AAA) remains a catastrophic event with high mortality. The critical time-sensitive challenge lies not in diagnosing rupture in a patient already in shock, but in identifying the hemodynamically stable patient whose aneurysm is with impending rupture: representing a true “ticking bomb” that demands emergency intervention within a few hours (1). However, current assessment of these symptomatic yet stable patients depends on subjective interpretation of computed tomography angiography (CTA) images, leading to interpretive variability that may cause missed impending rupture signs, diagnostic delays, and misdiagnosis rates approaching 30% (2–5).

Artificial intelligence (AI) holds promise for objective image analysis in vascular medicine (6–8). However, existing models have primarily focused on the elective surveillance pathway, notably automating segmentation or predicting long-term rupture risk in asymptomatic patients (9–13). While valuable for outpatient surveillance, these models are ill-suited to the acute emergency department setting. They function as “black-box” systems, analyzing imaging data in isolation while overlooking key pre-intervention clinical biomarkers—inflammation, hemodynamic stress, and metabolic state—that clinicians routinely use to assess systemic condition.

To address this gap, we developed an interpretable multimodal deep learning framework for emergency triage of symptomatic AAA, hypothesizing that predicting impending rupture requires capturing the synergistic interaction between systemic physiology and focal wall morphology beyond simple data concatenation. To achieve this, we employ a novel bidirectional cross-attention (BCA) mechanism that enables dynamic, iterative interaction between modalities: the clinical context guides attention to relevant imaging regions, while the imaging findings guide the interpretation of clinical features (14). This design mirrors the integrative reasoning of emergency clinicians, who consider laboratory and imaging data in concert. Crucially, this explainable AI framework delivers anatomically plausible insights per prediction, supporting a rapid, data-driven system for accurate risk stratification in acute vascular care.

## Methods

### Patients

This retrospective cohort study was conducted at the General Hospital of the Northern Theater Command, where we screened 1,123 consecutive patients diagnosed with AAA from June 1, 2017, to June 1, 2025. The inclusion criteria were (1) symptomatic AAA, (2) availability of CTA, and (3) age 18 years or older. Exclusion criteria included prior aortic repair, unstable hemodynamics, acute aortic syndrome, and incomplete CTA data, yielding a final cohort of 263 eligible patients. The study involving human participants was approved by the Institutional Review Board of the General Hospital of the Northern Theater Command (Approval No. Y2025[511]) and conducted in accordance with the Declaration of Helsinki. Informed consent was waived due to the retrospective design and use of anonymized data.

To rigorously evaluate the generalizability of our model, we employed an internal temporal validation strategy. From the 263 eligible patients, those presenting in the final year (June 1, 2024, to June 1, 2025) were reserved as an independent internal temporal validation cohort (*n* = 33), who were excluded from all development phases and participated only in the final testing. The remaining patients from June 1, 2017, to May 31, 2024, served as the development cohort (*n* = 230).

**Figure 1:**
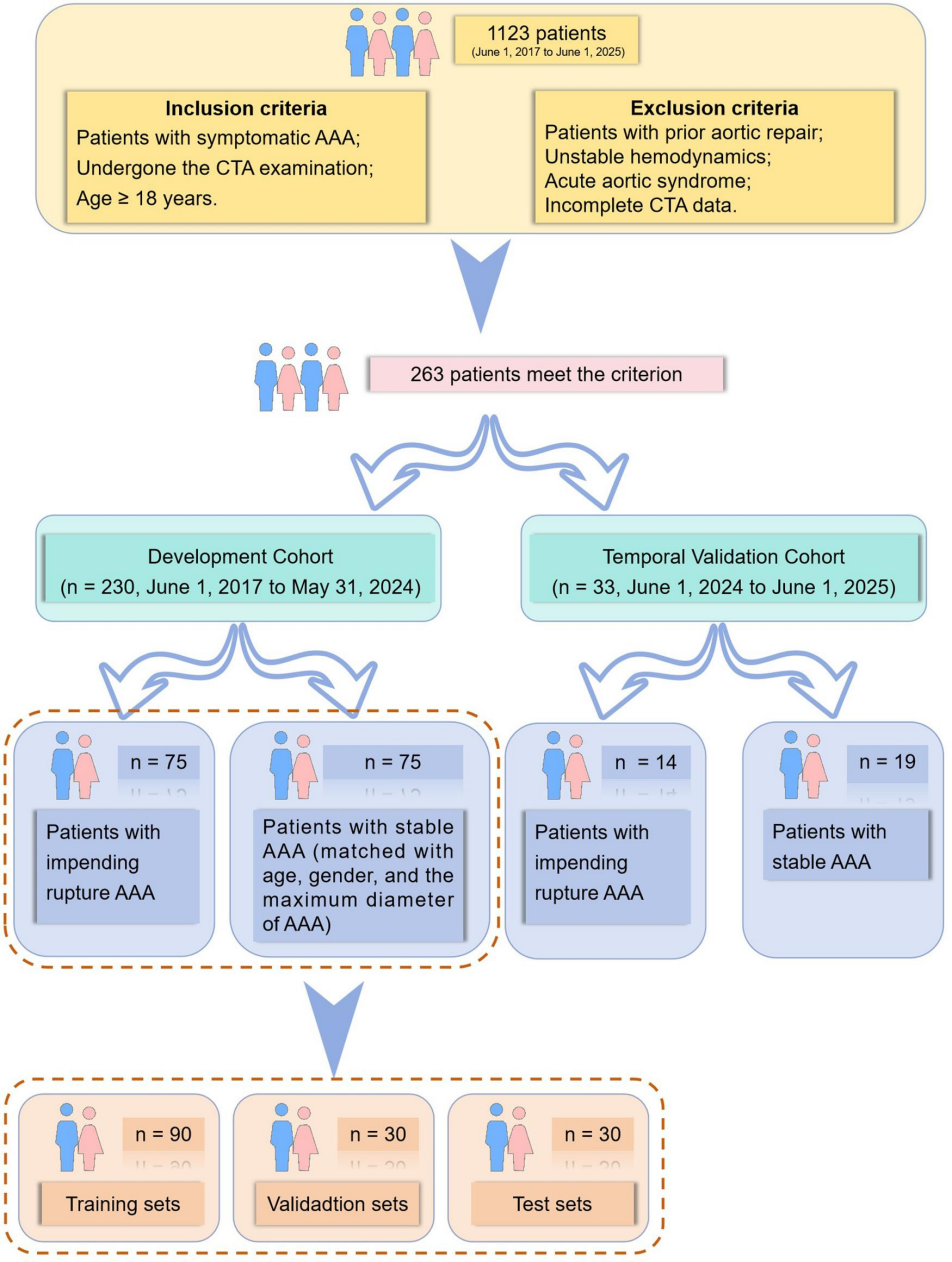
Flow chart of the study. AAA, abdominal aortic aneurysm.

To balance confounders between the impending rupture and stable groups within the development cohort, propensity score matching was performed. Patients were matched 1:1 on age, sex, and maximum aneurysm diameter using a caliper width of 0.2 (15), yielding 75 matched pairs (see Figure 1). All subsequent model training and internal validation were performed within this matched cohort.

### Definition

Impending rupture of an AAA was defined by the presence of a symptomatic AAA in hemodynamically stable patients, systolic blood pressure (SBP) > 90 mmHg without vasopressor support, accompanied by objective evidence of acute aortic wall failure necessitating emergency intervention within 24 hours. Cases were adjudicated by multidisciplinary consensus using a prespecified evidence hierarchy to ensure objectivity: (1) definitive high-risk CTA signs (e.g., crescent sign, discontinuous intimal calcification, focal aortic bleb); (2) CTA findings of contained rupture (e.g., perianeurysmal hematoma, draped aorta sign, focal wall discontinuity), with the latter superseding high-risk signs when present; and (3) intraoperative confirmation, reserved for cases where CTA was non-diagnostic but prior imaging revealed dynamic changes(e.g., rapid enlargement or thrombus evolution). The primary evidence category was recorded for each case to facilitate transparent endpoint delineation and subsequent evidence-based sensitivity analyses.

Clinical predictors were derived from the initial emergency department (ED) assessment to reflect the pre-intervention state and minimize confounding by treatment effects. Significant intervention was defined as any post-triage treatment that could alter core physiology, including intravenous fluids, blood transfusion, vasoactive medications, or anesthesia. Accordingly, the first recorded vital signs, including SBP and diastolic blood pressure (DBP), height and weight measurements, and laboratory values, were extracted. To enforce temporal validity, a structured audit categorized each patient according to definitive documentation: (1) strictly pre-intervention (sampling before any significant intervention), (2) unclear timing, or (3) post-intervention. Following this audit, no patients in the matched development cohort (*n* = 150) had unclear timing, and all post-intervention labs were excluded at the source and treated as missing values. Thus, the primary analysis is based on temporally valid, pre-intervention biomarkers. This end-to-end protocol was designed to prevent contamination of predictors by treatment responses. Detailed event sequences and timing categories are provided in Supplementary Table S1.

### Clinical Data Preprocessing and Feature Selection

Clinical data and CTA images from all 263 eligible patients were collected. Preprocessing and feature selection were performed on the development cohort (*n* = 230), while model training and validation were restricted to the propensity-matched sub-cohort (*n* = 150, 75 impending rupture, 75 stable). The matched cohort was randomly split 6:2:2 at the patient level into training, validation, and test sets to maintain independence.

Variables with >50% missing values were excluded. Missing proportions for retained features are detailed in Supplementary Table S3, with no significant association with the outcome (all *P* > 0.05). Among four imputation methods evaluated, k-nearest neighbors (KNN, k = 5) was selected for best preserving the original data distribution (16). Critically, the KNN imputer and the StandardScaler were fitted exclusively on the training set, then applied to the validation, test, and temporal validation sets. Following imputation, continuous features were standardized to zero mean and unit variance, and the outcome variable (stable vs. impending rupture) was binarized as 0 and 1.

To address multicollinearity, highly correlated features (Pearson | r | > 0.8) were removed prior to selection, retaining only the most clinically relevant from each correlated pair (17). A multi-algorithm feature selection framework was then applied using logistic regression, extreme gradient boosting (XGBoost), minimum redundancy maximum relevance (mRMR), gradient-boosted decision trees (GBDT), and random forest. Each algorithm ranked features by intrinsic importance, with the top six from each method forming candidate subsets. Subset stability was assessed by selection frequency across 1000 bootstrap iterations to mitigate overfitting. The optimal subset was determined using a composite score balancing predictive performance and robustness: Composite Score = 0.6 × AUC + 0.4 × Bootstrap Stability, where AUC denotes the area under the receiver operating characteristic (ROC) curve. This weighting prioritizes discriminative power while penalizing instability, a key consideration for generalizability in small-to-moderate cohorts. For each candidate set, an L2-regularized logistic regression classifier was trained, and its validation AUC was recorded. Calibration was additionally assessed using the Hosmer-Lemeshow goodness-of-fit test. The candidate set with the highest composite score was selected as the final feature subset, thereby balancing discriminative power, stability, and clinical interpretability.

### CTA acquisition and preprocessing

CTA images were retrieved from the Picture Archiving and Communication System. Scans were obtained using CT scanners from four manufacturers (Neusoft, Philips Healthcare, GE Healthcare, and Canon Medical Systems; see Supplementary Table S1. To preserve contrast information consistent with radiologic interpretation, native Hounsfield unit (HU) values were windowed using a standard arterial phase vascular preset (window width: 350 HU; window level: 40 HU), converting images to 8-bit grayscale (pixel range 0–255). Although acquisition parameters varied by device and evolved over the study period, all examinations met diagnostic AAA standards. Common specifications included detector collimation of 128 × 0.625 mm, gantry rotation time of 270 ms, automated tube voltage selection (100–120 kVp), and tube current modulation (500–700 mAs). Image acquisition employed retrospective electrocardiogram gating with intravenous iodinated contrast (Ioversol, 320 mgI/mL) administered at weight-adjusted doses of 1–1.5 mL/kg and injection rates of 4–6 mL/s.

All images were reconstructed during the arterial phase, at the time of peak aortic enhancement. A consistent reconstruction protocol was applied across all scanners, including a section thickness of 0.625 mm, a reconstruction increment of 0.5 mm, and reconstruction centered at 75% of the R–R interval.

To address scanner heterogeneity, standardized image preprocessing was applied during model development. Intensity values were normalized using ImageNet mean and standard deviation, and data augmentation (random rotation, flipping, color jitter, Gaussian blur, MixUp, CutMix) was applied during training (Supplementary Table S4).

For consistent anatomical input, each anonymized CTA volume underwent standardized preprocessing. Two board-certified radiologists independently marked the axial slices from the caudal renal artery origin to the aortic bifurcation. All contiguous slices between these landmarks were automatically included as the volume of interest (VOI), without manual aortic segmentation. This initialization required approximately 44 seconds (range 31–59) per case (Supplementary Tables S5, S6). The VOI was then processed through an automated pipeline involving resizing, normalization, augmentation, and converted to a three-channel RGB format for model compatibility. Thus, the core multimodal model operates end-to-end, generating a prediction directly from the defined VOI and clinical data. It is important to note that the current clinical pipeline retains this manual initialization step.

### Multimodal model architecture

**Figure 2:**
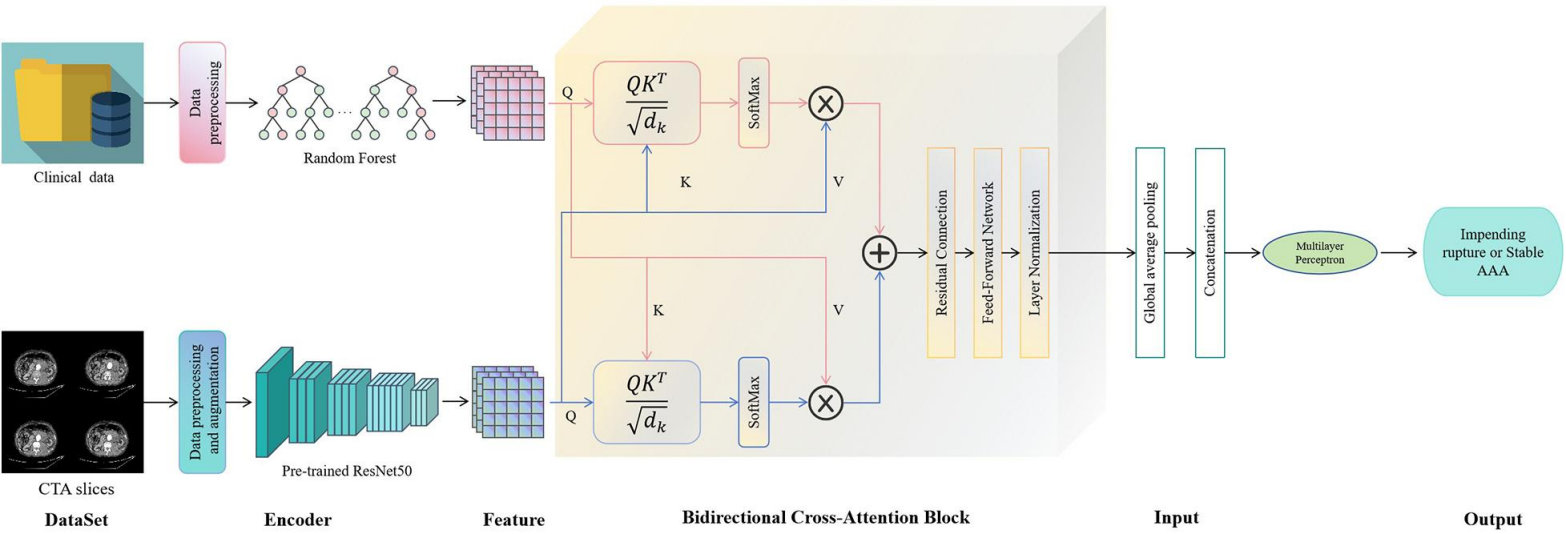
Overall framework of the multimodal model. AAA, abdominal aortic aneurysm; CTA, computed tomography angiography.

We proposed a multimodal fusion model integrating CTA slices with clinical features via a BCA mechanism for binary classification (18–20). The architecture comprised three core components: an image feature encoder, a clinical feature encoder, and a BCA fusion module (Figure 2).

For the image encoder, we evaluated multiple architectures commonly used in medical image analysis, including convolutional (AlexNet, ResNet 18/50/101, DenseNet121, VGG16) and transformer-based (ViT-B/16, and MedViT) networks. For each patient, 128 axial slices were obtained between the renal arteries and the aortic bifurcation using fixed-interval sampling. Each slice was resized to 224 × 224 pixels and converted to RGB format. The slice sequence was passed through the backbone to extract slice-level features, which were then projected into a shared 512-dimensional latent space via a projection head. This head comprised a linear layer (backbone output to 2048), followed by Gaussian Error Linear Unit (GELU) activation, layer normalization, dropout (rate = 0.3), a second linear layer (2048 to 512), and then GELU activation, layer normalization, and dropout.

For the clinical feature encoder, the six features selected by random forest were Z-score normalized and passed through a two-layer multilayer perceptron (MLP), projecting them into the same 512-dimensional latent space as the image features. The first layer mapped six features to 256 dimensions, followed by GELU activation and layer normalization. The second layer mapped 256 to 512 dimensions, again with GELU activation and layer normalization. Dropout (0.3) was applied.

The BCA module fuses modalities by enabling mutual interaction between the image feature sequence (slice embeddings) and the clinical feature vector (single embedding). The module contained four sequential blocks, each employing multi-head attention (8 heads). Within each BCA block, operations included: (1) layer normalization of both modalities; (2) image-to-clinical attention, allowing clinical context to weight relevant imaging slices; (3) clinical-to-image attention, infusing the slice-level features with clinical context; and (4) a feed-forward network with residual connections for feature refinement. After the four blocks, mean pooling was applied to the refined image feature sequence. The pooled image embedding and refined clinical embedding were then concatenated to form a unified multimodal representation, which was fed into a final three-layer MLP classifier (dimensions 1024, 512, and 2, with GELU activation, layer normalization, and dropout) to generate the binary prediction (impending rupture vs. stable).

For image preprocessing, all input images were resized to 224 × 224 pixels and normalized using the ImageNet mean and standard deviation [mean [0.485, 0.456, 0.406], standard deviation [0.229, 0.224, 0.225]], with center cropping applied to preserve anatomical integrity. Extensive data augmentation was employed to improve robustness, including random resized cropping, flipping, rotation (±20°), color jitter, Gaussian blur, random erasing, and MixUp and CutMix (21). Detailed augmentation parameters and hyperparameter configurations are provided in the Supplementary Tables S7, S8. A global random seed of 42 was set for all experiments. Model training was optimized using Focal Loss (*α*=0.75, *γ*=2) with the AdamW optimizer incorporating lookahead (22), a learning rate of 3e-5, weight decay of 1e-5, and a OneCycleLR scheduler. Early stopping (patience=30) and exponential moving average (decay=0.999) were applied to stabilize training. To manage GPU memory constraints, we used automatic mixed-precision training, gradient accumulation (steps=4), and gradient checkpointing. An auxiliary loss (weight=0.3) was also incorporated to enhance robustness. For evaluation, test-time augmentation (TTA) was applied using flipping, rotation (±15°), and affine transformations, with predictions averaged across augmented versions. The model was implemented in PyTorch 2.7.0 and trained on a single NVIDIA GeForce RTX 4090 GPU (24 GB VRAM).

### Comparative experiment

To comprehensively evaluate the proposed framework, covering technical architecture and clinical relevance, we performed multiple comparative analyses. Technical ablation studies included: (1) comparing image preprocessing strategies (three-channel ImageNet-normalized pipeline vs. CT-aware single-channel Z-score normalized approach); (2) comparing deep learning backbones for image encoding [AlexNet, VGG16, ResNet (18/50/101), DenseNet-121, ViT-B/16, and MedViT]; (3) replacing the BCA module with alternative attention mechanisms (multi-head self-attention, squeeze-and-excitation, gated attention); and (4) evaluating early-stopping patience (10, 20, 30, and 40 epochs) on model performance. To establish clinically meaningful benchmarks, we developed two baseline models reflecting current practice: a simple clinical rule-based model using traditional variables including maximum diameter, SBP, hemoglobin, C-reactive protein, and random blood glucose (RBG), and a structured CTA sign model based on logistic regression incorporating key morphological features selected via random forest (sacciform AAA, maximum mural thrombus area, minimum lumen diameter, and the maximum and minimum diameters of the left external iliac artery). Discriminative performance of the proposed multimodal model and these two baselines was compared using the DeLong test for paired AUCs. All model development was performed on the training set.

### Model interpretability and visualization

To provide qualitative insight into the model's decision-making, we used Gradient-weighted Class Activation Mapping (Grad-CAM) to visualize image regions most influential during classification (23, 24). This technique identifies regions statistically associated with the model output, though it does not establish causal determinants of pathology. The core classification model was trained end-to-end on image volumes and clinical data without reliance on manual annotations; thus, the Grad-CAM visualizations reflect data-driven patterns learned by the model. For clinically efficient evaluation, Grad-CAM was applied to a representative subset of 20 equally spaced axial slices per patient. In the independent temporal validation cohort (*n* = 33), two senior vascular radiologists, blinded to model predictions and final diagnoses, independently performed a structured plausibility assessment (25) based on two explicit criteria: (a) anatomical coherence, whether primary heatmap activation localized to the aortic wall, lumen, or immediate periaortic space; and (b) spatial relevance, whether activation overlapped with regions typically scrutinized for instability signs (e.g., focal wall thickening, hematoma, or discontinuity). The goal was to assess face validity, whether the model's focus was generally anatomically plausible, rather than to validate specific pathophysiological findings.

### Statistics

Continuous variables were reported as medians with interquartile ranges (IQR) and compared using the Mann-Whitney U test; categorical variables as counts (percentages) using chi-square or Fisher's exact test. Propensity score matching controlled for confounders between groups. Model performance was evaluated using the AUC of ROC. To assess clinical utility, we reported sensitivity, specificity, positive predictive value (PPV), and negative predictive value (NPV) using a fixed 0.5 threshold applied uniformly across all cohorts without tuning. The 95% confidence intervals (CI) for all metrics were derived from 1000 nonparametric bootstrap replicates. Calibration was assessed using calibration plots, Brier scores, and expected calibration error (ECE), with clinical utility further evaluated via decision curve analysis (DCA). Three prespecified sensitivity analyses examined robustness: (1) stratification by evidence strength (imaging-objective evidence, high-risk CTA signs only, hard-objective evidence, and contained rupture on CTA only); (2) complete-case analysis (*n* = 109) comparing baseline characteristics between complete-case and imputation-required subsets to assess selection bias; and (3) assessments of temporal drift and scanner heterogeneity. All included patients had confirmed pre-intervention sampling; post-intervention results were excluded at source. Performance metrics are reported separately for the development and internal temporal validation cohorts, with detailed formulations provided in Supplementary Materials. Analyses were performed using IBM SPSS version 26 (Armonk, NY) and Python 3.12.9.

## Results

### Baseline characteristics

The study included 150 propensity-matched patients with infrarenal AAAs (75 impending rupture and 75 stable). The cohort was predominantly male (89.3%) with a median age of 69 years (IQR 64–73). Compared to the stable controls, impending rupture patients exhibited lower SBP (130 vs. 140 mmHg, *P* = 0.015), DBP (75 vs. 90 mmHg, *P* < 0.001), and hemoglobin (110 vs. 126 g/L, *P* < 0.001), along with higher white blood cell (WBC) count (12.4 vs. 7.7 × 10⁹/L, *P* < 0.001), and random blood glucose (RBG, 8.3 vs. 6.4 mmol/L, *P* = 0.004). The impending rupture group also had lower rates of smoking history (12% vs. 28%, *P* = 0.014), drinking history (6.7% vs. 18.7%, *P* = 0.027), hypertension (20% vs. 36%, *P* = 0.029), prior surgery (6.7% vs. 17.3%, *P* = 0.044), and aspirin therapy (8% vs. 24%, *P* = 0.008). No other clinical characteristics differed significantly (all *P* *>* 0.05). Complete data are presented in Table 1 and Supplementary Table S9**.**

**Table 1:** Comparison of baseline characteristics between the stable group and the impending rupture group.

Characteristic	Overall	Stable	Impending rupture	Statistic^1^	p-value^1^
	*N* = 150	*N* = 75	*N* = 75		
Age	69 (64, 73)	68 (63, 73)	70 (65, 74)	2,502.00	0.243
Male, n (%)	134 (89.3%)	68 (90.7%)	66 (88.0%)	0.28	0.597
Smoking, n (%)	30 (20.0%)	21 (28.0%)	9 (12.0%)	6	0.014
Drinking, n (%)	19 (12.7%)	14 (18.7%)	5 (6.7%)	4.88	0.027
Hypertension, n (%)	42 (28.0%)	27 (36.0%)	15 (20.0%)	4.76	0.029
Diabetes, n (%)	10 (6.7%)	8 (10.7%)	2 (2.7%)	3.86	0.05
CHD, n (%)	25 (16.7%)	15 (20.0%)	10 (13.3%)	1.2	0.273
Prior Stroke, n (%)	17 (11.3%)	10 (13.3%)	7 (9.3%)	0.6	0.44
Prior surgery, n (%)	18 (12.0%)	13 (17.3%)	5 (6.7%)	4.04	0.044
SBP, mmHg	136 (120, 148)	140 (129, 156)	130 (105, 145)	1,249.50	0.015
DBP, mmHg	78 (72, 94)	90 (79, 100)	75 (68, 80)	1,518.00	<0.001
Laboratory examination					
Hemoglobin, g/L	116 (102, 136)	126 (112, 148)	110 (93, 127)	2,068.50	<0.001
WBC,10^9^/L	9.7 (7.1, 13.9)	7.7 (5.4, 9.3)	12.4 (9.4, 15.6)	590	<0.001
Platelet,10^9^/L	187 (159, 222)	178 (146, 215)	191 (164, 232)	1,217.00	0.112
CRP, mg/L	12 (6, 44)	10 (4, 22)	15 (7, 51)	597.5	0.143
Fibrinogen, g/L	3.31 (2.73, 4.39)	3.33 (2.75, 4.33)	3.30 (2.56, 4.51)	1,466.00	0.919
D-Dimer, mg/dL	5 (2, 12)	4 (1, 12)	6 (3, 13)	1,277.00	0.43
RBG, mmol/L	7.0 (5.6, 9.9)	6.4 (5.5, 7.6)	8.3 (5.7, 13.2)	553.5	0.004
TC, mmol/L	4.27 (3.72, 5.00)	4.38 (3.96, 5.44)	3.87 (3.52, 4.45)	243	0.09
Triacylglycerol, mmol/L	1.26 (1.00, 2.04)	1.36 (1.00, 2.18)	1.19 (1.03, 1.52)	214	0.382
LDL-C, mmol/L	2.52 (2.17, 2.98)	2.57 (2.17, 3.06)	2.47 (2.19, 2.55)	217.5	0.327
Creatinine, mmol/L	89 (66, 136)	90 (65, 111)	89 (67, 156)	1,244.00	0.444
Prior medications					
Statins, n (%)	21 (14.0%)	13 (17.3%)	8 (10.7%)	1.38	0.239
Aspirin, n (%)	24 (16.0%)	18 (24.0%)	6 (8.0%)	7.14	0.008

Categorical variables are reported as frequency and percentage (n, %).

Continuous variables are reported as median and interquartile range med(Q1-Q3).

CHD, coronary atherosclerotic heart disease; SBP, systolic blood pressure; DBP, diastolic blood pressure; WBC, white blood cell; CRP, C-reactive protein; RBG, random blood glucose; TC, total cholesterol; LDL-C, low-density lipoprotein cholesterol; AAA, abdominal aortic aneurysm.

Morphological analysis of CTA images revealed distinct characteristics between the two groups. No significant differences were observed in the aneurysm length, calcification, or ulcer, intramural hematoma, and intraluminal thrombus (all *P* > 0.05). However, the impending rupture group showed a higher proportion of sacciform aneurysms (40.0% vs. 13.3%, *P* < 0.001), and larger minimum intraluminal diameter (25 vs. 21 mm, *P* = 0.041), but smaller diameters across multiple iliac artery segments (e.g., minimum left common iliac artery: 12.4 vs. 14.2 mm, *P* = 0.010). No other morphological features differed significantly between groups (Table 2).

**Table 2:** Comparison of baseline CTA characteristics between the stable group and the impending rupture group.

Characteristic	Overall	stable	Impending rupture	Statistic	p-value
	*N* = 150	*N* = 75	*N* = 75		
Neck length, mm	22 (11, 34)	23 (12, 34)	21 (10, 34)	3060.50	0.352
Aneurysm length, mm	104 (82, 125)	105 (86, 125)	97 (80, 129)	3102.00	0.277
Maximum diameter of AAA, mm	49 (41, 61)	49 (41, 61)	50 (40, 61)	2906.50	0.725
Sacciform	40 (26.7%)	10 (13.3%)	30 (40.0%)	13.64	<0.001
Abdominal aorta Ulcer	96 (64.0%)	46 (61.3%)	50 (66.7%)	0.46	0.496
Abdominal aorta intramural hematoma	21 (14.0%)	8 (10.7%)	13 (17.3%)	1.38	0.239
Abdominal aorta dissection	11 (7.3%)	6 (8.0%)	5 (6.7%)	0.10	0.754
Abdominal aorta calcification	89 (59.3%)	39 (52.0%)	50 (66.7%)	3.34	0.067
ILT	124 (82.7%)	65 (86.7%)	59 (78.7%)	1.67	0.196
Mean neck diameter, mm	23 (20, 25)	23 (21, 26)	22 (18, 25)	3338.50	0.048
Minimum aneurysm intraluminal diameter, mm	23 (17, 28)	21 (16, 27)	25 (20, 29)	2267.00	0.041
Suprarenal angulation, degree	167 (150, 175)	167 (153, 175)	169 (148, 175)	2813.00	>0.999
Infrarenal Angulation, degree	25 (10, 51)	26 (11, 52)	24 (9, 46)	3021.00	0.434
Maximum diameter of LCIA, mm	18 (15, 23)	18 (15, 24)	17 (14, 21)	3283.50	0.077
Minimum diameter of LCIA, mm	13.2 (10.7, 16.2)	14.2 (11.4, 17.3)	12.4 (10.0, 15.4)	3495.00	0.010
Maximum diameter of RCIA, mm	19 (15, 25)	20 (16, 27)	17 (15, 22)	3441.50	0.018
Minimum diameter of RCIA, mm	13.7 (11.2, 16.5)	14.6 (11.4, 17.6)	13.2 (10.1, 15.6)	3531.50	0.007
Maximum diameter of LEIA, mm	9.49 (8.30, 10.60)	10.00 (8.80, 10.80)	8.81 (8.00, 10.10)	3636.00	0.002
Maximum diameter of REIA, mm	9.70 (8.50, 11.30)	10.50 (8.89, 11.90)	9.30 (7.80, 10.40)	3675.50	0.001
Ratio of ILT area	0.52 (0.21, 0.70)	0.57 (0.31, 0.77)	0.50 (0.13, 0.65)	3272.50	0.083

CTA, computed tomography angiography; AAA, abdominal aortic aneurysm; ILT, intraluminal thrombus; LCIA, left common iliac artery; RCIA, right common iliac artery; LEIA, left external iliac artery; REIA, right external iliac artery.

### Composition of the impending rupture cohort

Per the prespecified adjudication process, the primary evidence category was recorded for each of the 75 impending rupture cases. Cases were stratified into two categories (Table 3): hard-objective evidence (contained rupture on CTA or intraoperative confirmation, *n* = 19, 25.3%) and high-risk CTA signs only (e.g., crescent sign, calcification discontinuity, aortic bleb, *n* = 56, 74.7%). No case was classified based on clinical judgment alone without objective correlates. This transparent stratification defines the endpoint composition and supports the evidence-based sensitivity analyses as follows.

**Table 3:** Evidence composition of impending rupture cases in the development and temporal validation cohorts.

Evidence Category	Development cohort (*N* = 75)	Development cohort Test set (*N* = 15)	Temporal Validation cohort test set (*N* = 14)
Hard-objective evidence	19 (25.33%)*	4 (26.67%)*	5 (35.71%)*
Contained rupture on CTA	12 (16.00%)	3 (20.00%)	4 (28.57%)
Draped aorta sign	5 (6.67%)	1 (6.67%)	1 (7.14%)
Focal aortic wall discontinuity	4 (5.33%)	2 (13.33%)	1 (7.14%)
Perianeurysmal hematoma	7 (9.33%)	2 (13.33%)	2 (14.29%)
Intraoperative confirmation	7 (9.33%)	1 (6.67%)	1 (7.14%)
Aortic wall perforation with contrast extravasation	3 (4.00%)	0 (0.00%)	1 (7.14%)
Organized hematoma	3 (4.00%)	1 (6.67%)	0 (0.00%)
Retroperitoneal hematoma	1 (1.33%)	0 (0.00%)	0 (0.00%)
High-risk CTA signs	56 (74.67%)*	11 (73.33%)	9 (64.29%)
Focal wall discontinuity of circumferential calcifications	29 (38.67%)	7 (46.67%)	4 (28.57%)
Crescent sign	21 (28.00%)	2 (13.33%)	3 (21.43%)
Aortic bleb	9 (12.00%)	2 (13.33%)	2 (14.29%)
Clinical attribution alone	0(0.00%)	0(0.00%)	0(0.00%)

CTA, Computed tomography angiography.

*Some cases exhibited multiple signs; therefore, the sum of percentages may exceed 100%.

### Clinical feature selection

**Figure 3:**
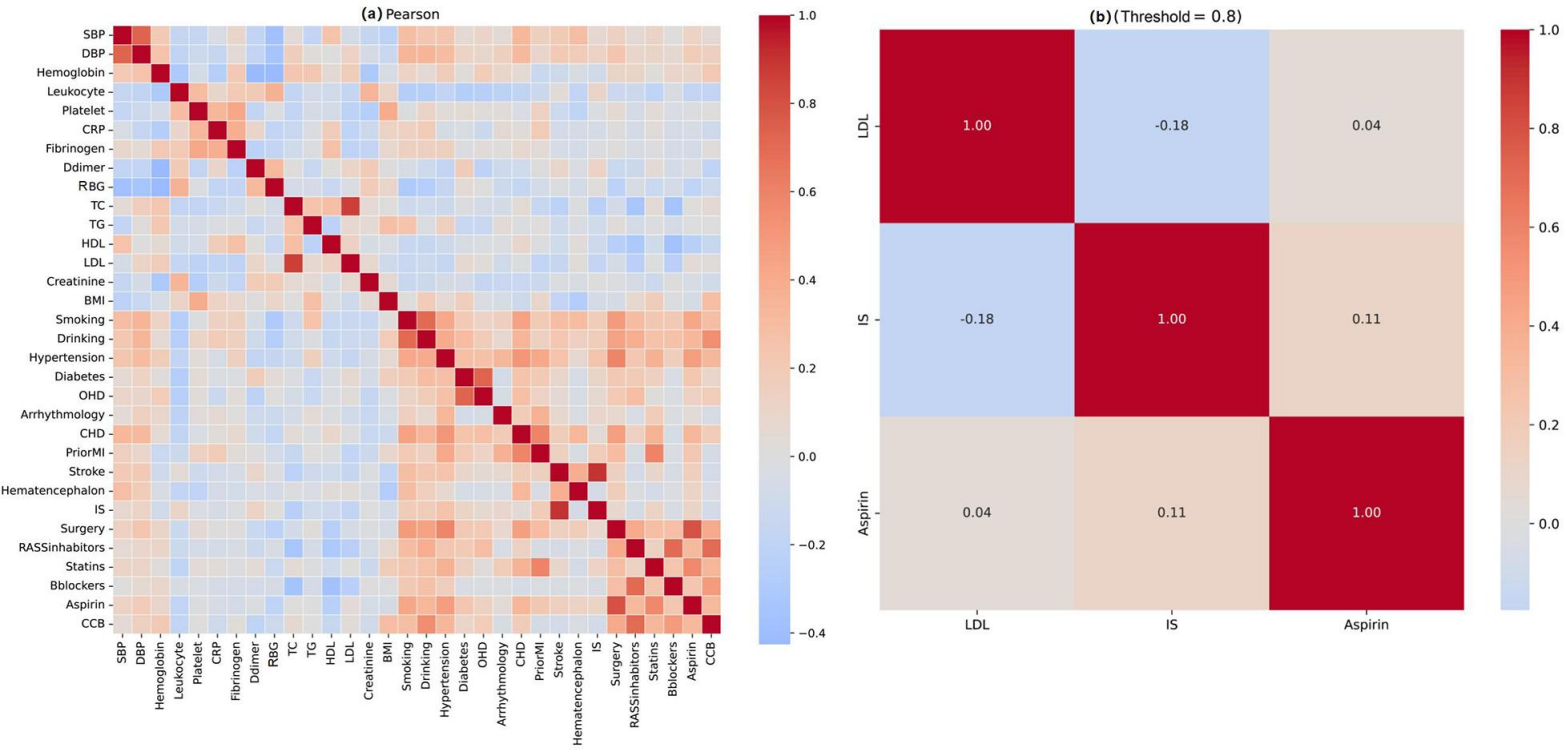
Pearson correlation analysis matrix. **(a)** Correlation analysis matrix of all clinical features; **(b)** strongly correlated variables.

**Figure 4:**
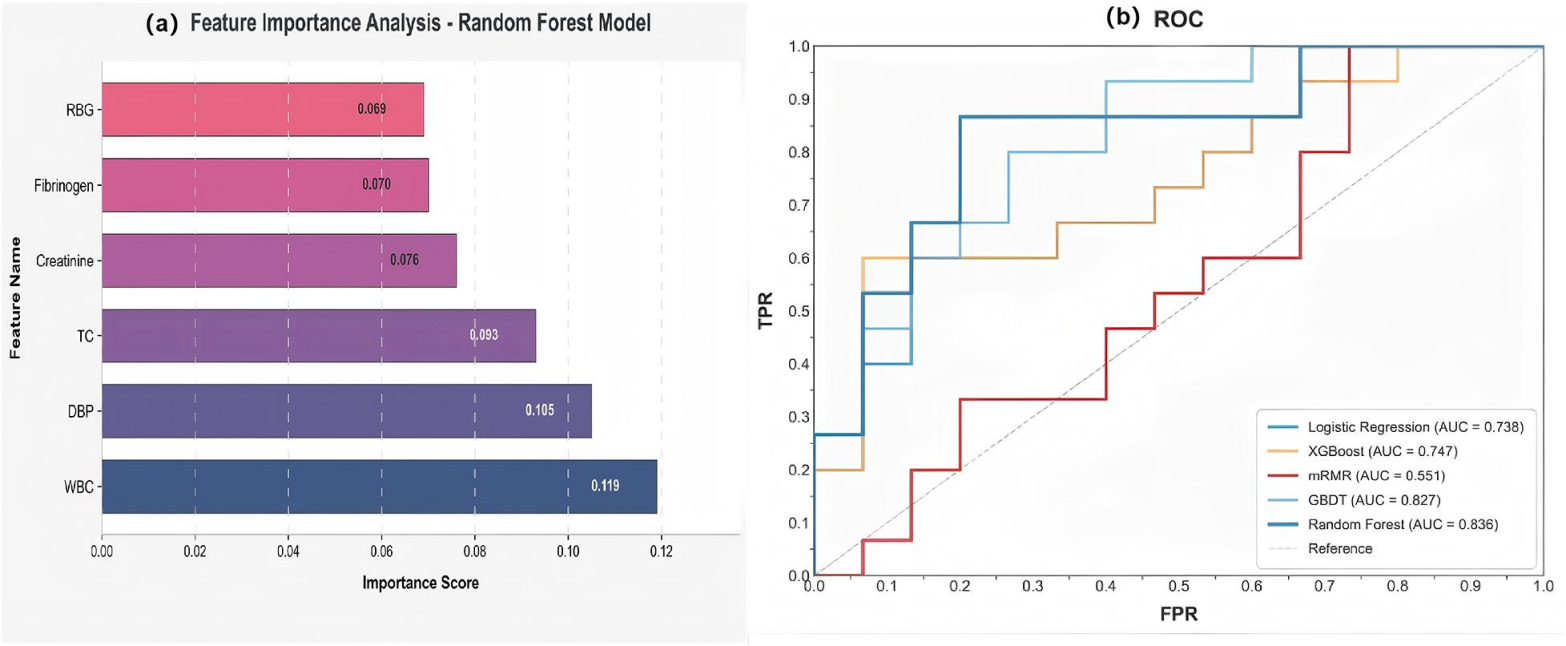
The clinical feature selection based on five machine learning models. **(a)** The importance ranking of the top six variables based on Random Forest; **(b)** receiver operating characteristic curves of the clinical feature selection model. RBG, random blood glucose; TC, total cholesterol; DBP, diastolic blood pressure; WBC, white blood cell; AUC, area under the curve; TPR, true positive rate; FPR, false positive rate.

The dataset was divided into training (*n* = 90), validation (*n* = 30), and test (*n* = 30) sets (Supplementary Table S10). Missing values were imputed via KNN (k = 5), with post-imputation distributions verified (Supplementary Tables S11, S12). To address multicollinearity, three highly correlated variables (|r| > 0.8) were excluded based on Pearson analysis (Figure 3): low-density lipoprotein cholesterol (correlated with total cholesterol), prior ischemic stroke (with coronary disease), and aspirin therapy (with platelet count). Five feature selection methods were evaluated: logistic regression, XGBoost, mRMR, GBDT, and random forest. The six-feature subset from random forest achieved the highest composite score (0.724), with a validation AUC of 0.836 (95% CI: 0.782–0.887) and bootstrap selection frequency of 55.55% (Supplementary Table S13). Hosmer-Lemeshow testing confirmed excellent calibration (*χ*^2^ = 2.823, *P* = 0.945). Final selected features were WBC count, DBP, total cholesterol, creatinine, fibrinogen, and RBG (Figure 4). Results from the other four selection methods are provided in Supplementary Figures S1–S4.

### Multi modal feature fusion model

**Figure 5:**
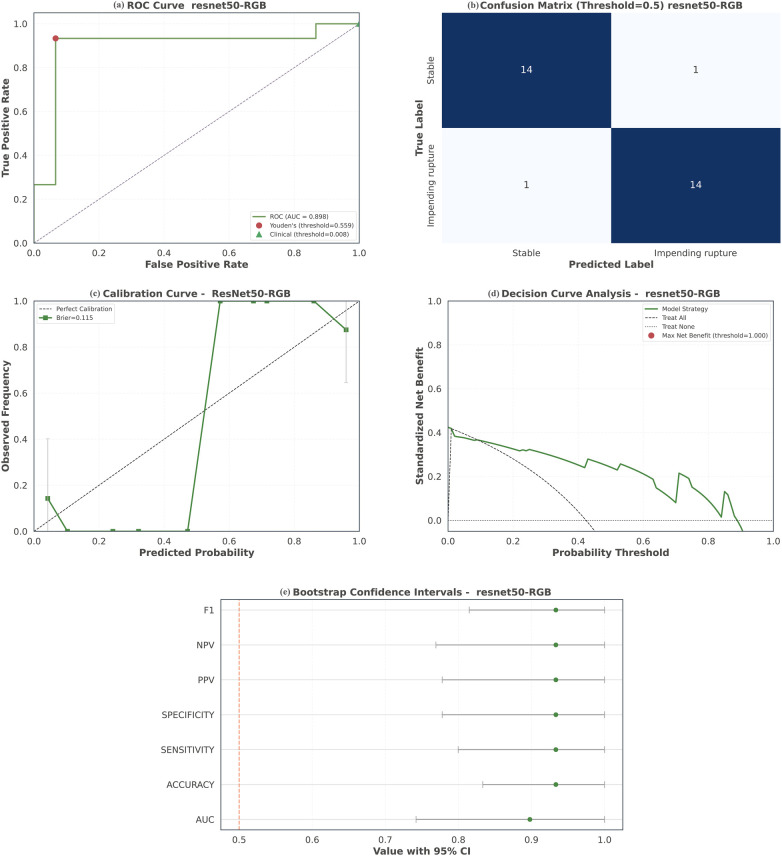
Performance evaluation of the proposed multimodal model in the development cohort. **(a)** ROC curve of the multimodal model; **(b)** confusion matrices of the multimodal model; **(c)** calibration curve; **(d)** decision curve analysis; **(e)** bootstrap confidence intervals. ROC, receiver operating characteristic; AUC, area under the curve.

Axial CTA images from the renal arteries to the aortic bifurcation were manually delineated by expert radiologists and served as input to the multimodal fusion model. Among eight evaluated backbones, ResNet-50 was selected as the optimal encoder. On the development test set, the ResNet50-based multimodal model achieved strong discriminative performance for identifying impending rupture, with an AUC of 0.898 (Table 4). Crucially, for emergency triage where safely ruling out rupture is paramount, the model attained 93.3% sensitivity and 93.3% NPV at its default threshold, meaning a “low-risk” prediction would miss only 6.7% of true ruptures and providing a quantifiable safety margin for clinical decision-making. Model predictions were well calibrated, with a Brier score of 0.115 and an ECE of 0.196, indicating accurate risk estimation across intermediate probabilities. This ECE reflects an average deviation of 19.6 percentage point deviation between predicted and observed outcomes, an acceptable level given that risk stratification, rather than precise probability estimation, is the primary goal in emergency triage. Figure 5 provides a multi-panel visualization of validation results, integrating the ROC curve, confusion matrix, calibration curve, DCA, and bootstrap CI comparisons. Slight overestimation in the highest risk decile reflects a “safety-first” bias appropriate for this setting. DCA confirmed clinical utility, demonstrating superior net benefit versus “intervene-all” and “intervene-none” strategies across clinically relevant risk thresholds (e.g., 10% and 70%). The ResNet-50-based model outperformed all other tested backbones, as detailed in Table 4 and Supplementary Figures S5–S11. Corresponding training and validation loss curves appear in the Supplementary Figure S12.

**Table 4:** Performance analysis of the ResNet50 and other models to identify the impending rupture AAA.

Backbone	Accuracy	F1	AUC	Sensitivity	Specificity	PPV	NPV	Brier	ECE
AlexNet	0.733	0.765	0.844	0.867	0.6	0.684	0.818	0.194	0.172
ResNet18	0.8	0.786	0.853	0.733	0.867	0.846	0.765	0.139	0.158
ResNet50	0.933	0.933	0.898	0.933	0.933	0.933	0.933	0.115	0.196
ResNet101	0.8	0.786	0.836	0.733	0.867	0.846	0.765	0.175	0.219
DenseNet121	0.767	0.741	0.818	0.667	0.867	0.833	0.722	0.154	0.156
VGG16	0.8	0.769	0.88	0.667	0.933	0.909	0.737	0.164	0.196
ViT-B/16	0.733	0.733	0.84	0.733	0.733	0.733	0.733	0.173	0.149
MedViT	0.8	0.8	0.858	0.8	0.8	0.8	0.8	0.151	0.158

AAA, abdominal aortic aneurysm; AUC, area under the curve; PPV, positive predictive value; NPV, negative predictive value; ECE, expected calibration error.

### Experiments to explore model performance

We systematically evaluated key model components through controlled experiments. First, to address the effect of image intensity representation, we compared our standard three-channel ImageNet-normalized pipeline with a CT-aware single-channel alternative. The three-channel approach yielded marginally better performance in the development and temporal validation cohorts (Supplementary Figures S13, S14). As summarized in Table 5, an ablation study of attention mechanisms integrated with the ResNet-50 backbone demonstrated that our proposed BCA module outperformed multi-head self-attention, squeeze-and-excitation, and gated attention alternatives. Furthermore, we compared three slice-sampling strategies (fixed-interval, central region, and random sampling) under consistent experimental conditions. Fixed-interval sampling achieved the highest overall performance (Table 6). Moreover, evaluation of early stopping patience parameters identified a value of 30 as providing the optimal trade-off between training efficiency and model generalization (Table 7).

**Table 5:** Evaluation of different attention modules using CTA images to identify the impending rupture AAA.

Attentions	Accuracy	F1	AUC	Sensitivity	Specificity	PPV	NPV	Brier	ECE
ResNet50 + BCA	0.933	0.933	0.898	0.933	0.933	0.933	0.933	0.115	0.196
ResNet50 + GA	0.9	0.897	0.862	0.867	0.933	0.929	0.875	0.129	0.202
ResNet50 + SE	0.8	0.8	0.831	0.8	0.8	0.8	0.8	0.172	0.165
ResNet50 + MHSA	0.767	0.741	0.831	0.667	0.867	0.833	0.722	0.177	0.168

AAA, abdominal aortic aneurysm; AUC, area under the curve; PPV, positive predictive value; NPV, negative predictive value; ECE, expected calibration error; BCA, bidirectional cross-attention; GA, gated-attention; SE, squeeze-and-excitation module; MHSA, multi-head self-attention.

**Table 6:** Effect of slice sampling strategies on multimodal model performance.

Sampling strategy	Accuracy	F1	AUC	Sensitivity	Specificity	PPV	NPV	Brier	ECE
Fixed Interval Sampling	0.933	0.933	0.898	0.933	0.933	0.933	0.933	0.115	0.196
Random sampling	0.833	0.815	0.88	0.733	0.933	0.917	0.778	0.148	0.16
Central region sampling	0.867	0.857	0.889	0.8	0.933	0.923	0.824	0.134	0.171

AUC, area under the curve; PPV, positive predictive value; NPV, negative predictive value; ECE, expected calibration error.

**Table 7:** Comparison of early stopping strategy parameters for the multimodal model with ResNet50 backbone.

Patience	Accuracy	F1	AUC	Sensitivity	Specificity	PPV	NPV	Brier	ECE
10	0.767	0.759	0.764	0.733	0.8	0.786	0.75	0.211	0.247
20	0.833	0.815	0.813	0.733	0.933	0.917	0.778	0.159	0.160
30	0.933	0.933	0.898	0.933	0.933	0.933	0.933	0.115	0.196
40	0.8	0.769	0.84	0.667	0.933	0.909	0.737	0.167	0.187

AUC, area under the curve; PPV, positive predictive value; NPV, negative predictive value; ECE, expected calibration error.

### Comparative performance against pragmatic clinical baselines

**Figure 6:**
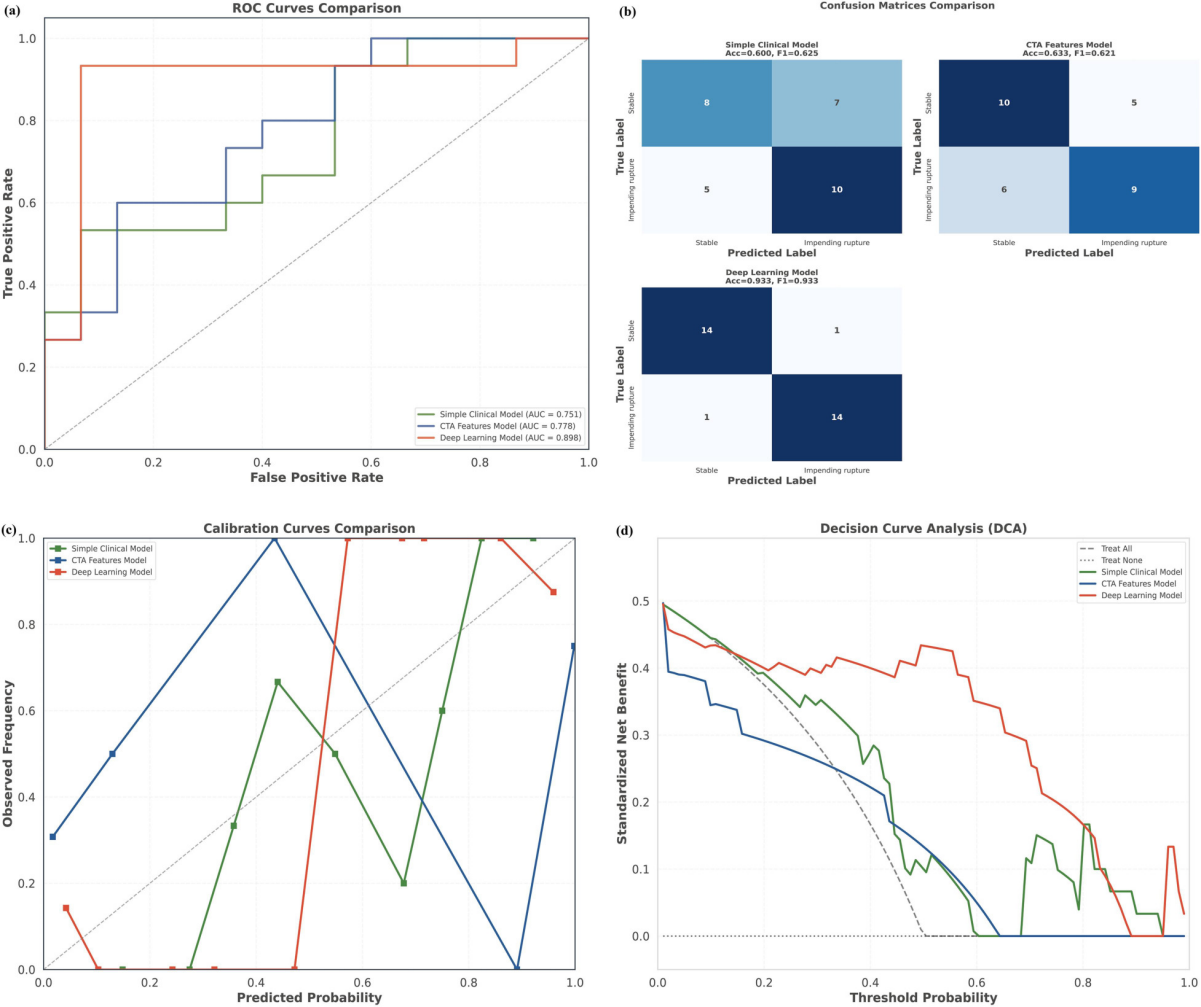
Comprehensive performance comparison of the triage models on the development test set. **(a)** ROC curves for the proposed deep learning, clinical rule-based, and CTA sign models; **(b)** confusion matrices; **(c)** calibration curves assessing the agreement between predicted probabilities and observed outcomes; **(d)** decision curve analysis. ROC, receiver operating characteristic; AUC, area under the curve.

To determine its incremental clinical utility, we compared our multimodal model with two pragmatic baselines mirroring current practice: a clinical-rule model and a structured CTA-sign model. The multimodal model yielded a higher AUC (0.898) than the clinical-rule (0.751) and CTA-sign (0.778) models (Figure 6, Table 8). Although pairwise AUC differences were not statistically significant by the DeLong test (both *P* > 0.05; Supplementary Table S14), the multimodal model demonstrated substantially superior performance on key metrics relevant to emergency triage. At the default threshold of 0.5, it achieved a sensitivity of 93.3% and an NPV of 93.3%, markedly exceeding the 61.5–62.5% observed with the baselines. Furthermore, the model exhibited improved calibration, with a Brier score of 0.115 and an expected ECE of 0.196. These metrics were substantially lower than the Brier scores of 0.210 and 0.312 for the clinical and CTA models, respectively (Table 8), indicating its probability estimates are more dependable for guiding clinical decisions. Collectively, these results underscore the clinically meaningful advantage over current pragmatic approaches, supporting both reliable identification of low-risk patients and efficient prioritization of high-risk cases for emergency intervention.

**Table 8:** Performance comparison of the multimodal deep learning model against clinical and imaging models for predicting impending rupture.

Modeling approach	Accuracy	F1	AUC	Sensitivity	Specificity	PPV	NPV	Brier	ECE
Simple Clinical Model	0.600	0.625	0.751 (0.560–0.903)	0.667	0.533	0.588	0.615	0.210	0.207
CTA Features Model	0.633	0.621	0.778 (0.607–0.928)	0.6	0.667	0.643	0.625	0.312	0.328
Deep Learning Model	0.933	0.933	0.898 (0.737–1.000)	0.933	0.933	0.933	0.933	0.115	0.196

AUC, area under the curve; PPV, positive predictive value; NPV, negative predictive value; ECE, expected calibration error; CTA, computed tomography angiography.

### Internal temporal validation cohort performance

**Figure 7:**
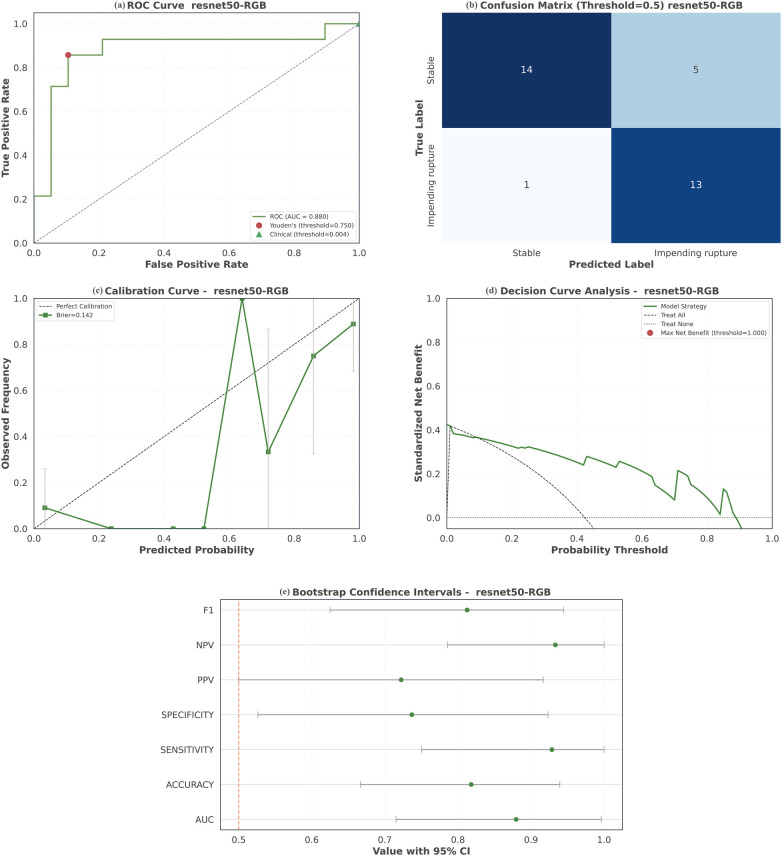
Performance of the multimodal model on internal temporal validation. **(a)** ROC curve of the multimodal model; **(b)** confusion matrices of the multimodal model; **(c)** calibration curve; **(d)** decision curve analysis; **(e)** bootstrap confidence intervals. ROC, receiver operating characteristic; AUC, area under the curve.

In an internal temporal validation cohort of 33 symptomatic AAA patients (78.8% male, mean age 74 years), 14 had impending rupture and 19 were stable. Among the 14 impending rupture cases, 9 were identified by high-risk CTA signs, 4 by contained rupture, and 1 by intraoperative confirmation. The model demonstrated strong performance: AUC 0.880, accuracy 81.8% (F1-score 0.812), sensitivity 92.9%, specificity 73.7%, with PPV and NPV of 70.6% and 87.5%, respectively, supporting reliable low-risk stratification, detailed in Figure 7. Calibration remained acceptable (Brier score 0.142; ECE 0.160), indicating potential utility in emergency settings. Time-breakdown analysis (Supplementary Table S15) revealed a median core inference time of 0.026 s per patient, increasing to 1.06 s with test-time augmentation. When full explainability, including Grad-CAM, was required, the total median time rose to 8.20 s. For rapid triage without visualization, the model delivers a prediction within ∼1.06 s, underscoring its suitability for real-time emergency decision-making.

### Sensitivity analysis across evidence-based subsets

To evaluate model robustness across levels of evidence certainty, we performed sensitivity analyses on nested subsets of increasing stringency (Table 9 and Supplementary Figures S15, S16). After excluding the single intraoperative-only case per cohort (imaging-objective subset), performance remained robust (AUCs 0.890 and 0.887), confirming that predictions were not driven by surgical decisions. To address potential circularity from including high-risk CTA signs in the endpoint definition, we analyzed the hard-objective evidence subset (contained rupture or intraoperative confirmation), excluding cases defined solely by high-risk signs. Here, the model maintained reasonable performance (AUC 0.75 in both cohorts), with 75–80% sensitivity and 74–93% specificity (Brier scores 0.15–0.19). This expected decline reflects inherent diagnostic difficulty and the smaller sample size of this advanced pathology subset rather than model failure, confirming predictions are not driven solely by endpoint-embedded high-risk signs. Conversely, in the high-risk CTA signs only subset, the model achieved excellent performance (AUCs 0.952–0.953, sensitivity 100%), underscoring its ability to detect endpoint-defining patterns.

**Table 9:** Sensitivity analysis of model performance across evidence-based subsets in the development test and temporal validation cohorts.

Cohort/Subset	Cases	Stable	Impending Rupture	AUC (95% CI)	Sensitivity	Specificity	PPV	NPV	Brier Score	ECE
Development Test Set										
Overall	30	15	15	0.898	0.933	0.933	0.933	0.933	0.115	0.196
Imaging objective evidence	29	15	14	0.89	0.929	0.929	0.929	0.933	0.119	0.178
Hard objective evidence	19	15	4	0.75	0.75	0.933	0.75	0.933	0.154	0.197
Contained rupture on CTA only	18	15	3	0.667	0.667	0.933	0.667	0.933	0.163	0.209
High-risk CTA signs only	26	15	11	0.952	1	0.933	0.917	1	0.095	0.197
Temporal Validation										
Overall	33	19	14	0.88	0.929	0.737	0.706	0.875	0.142	0.160
Imaging objective evidence	32	19	13	0.887	0.923	0.737	0.706	0.933	0.143	0.134
Hard objective evidence	24	19	5	0.75	0.8	0.737	0.444	0.933	0.191	0.22
Contained rupture on CTA only	23	19	4	0.737	0.75	0.737	0.375	0.933	0.194	0.214
High-risk CTA signs only	28	19	9	0.953	1	0.737	0.643	1	0.128	0.160

AUC, area under the curve; PPV, positive predictive value; NPV, negative predictive value; ECE, expected calibration error; CTA, computed tomography angiography.

### Sensitivity analysis on the complete-case subsets

To assess whether imputation influenced model performance, we repeated the analysis restricted to patients with complete pre-intervention data (*n* = 109), all of whom had confirmed pre-intervention sampling. This subset is hereafter referred to as the complete-case cohort. From this group, the complete-case test sets comprised 19 patients in the development cohort and 19 in the temporal validation cohort. As shown in Supplementary Table S16, this subset remained largely representative of the overall cohort in baseline characteristics, supporting generalizability. The model retained strong performance in these subsets (Table 10), with AUCs of 0.864 (development) and 0.857 (temporal validation), and specificity of 0.875 and 0.857, respectively. These findings confirm that the model's predictive ability is not contingent on imputation and remains robust in patients with complete-case data (Supplementary Figure S17), supporting its validity for early emergency triage.

**Table 10:** Model performance on the overall cohort and the complete-case subset with complete pre-intervention data.

Cohort/Subset	Cases	Stable	Impending Rupture	AUC (95% CI)	Sensitivity	Specificity	PPV	NPV	Brier Score	ECE
Development Test Set										
Overall	30	15	15	0.898	0.933	0.933	0.933	0.933	0.115	0.196
Complete-case	19	8	11	0.864	0.818	0.875	0.9	0.778	0.135	0.096
Temporal Validation										
Overall	33	19	14	0.88	0.929	0.737	0.706	0.875	0.142	0.160
Complete-case	19	7	12	0.857	0.833	0.857	0.909	0.75	0.149	0.131

AUC, area under the curve; PPV, positive predictive value; NPV, negative predictive value; ECE, expected calibration error.

### Domain shift analysis

**Figure 8:**
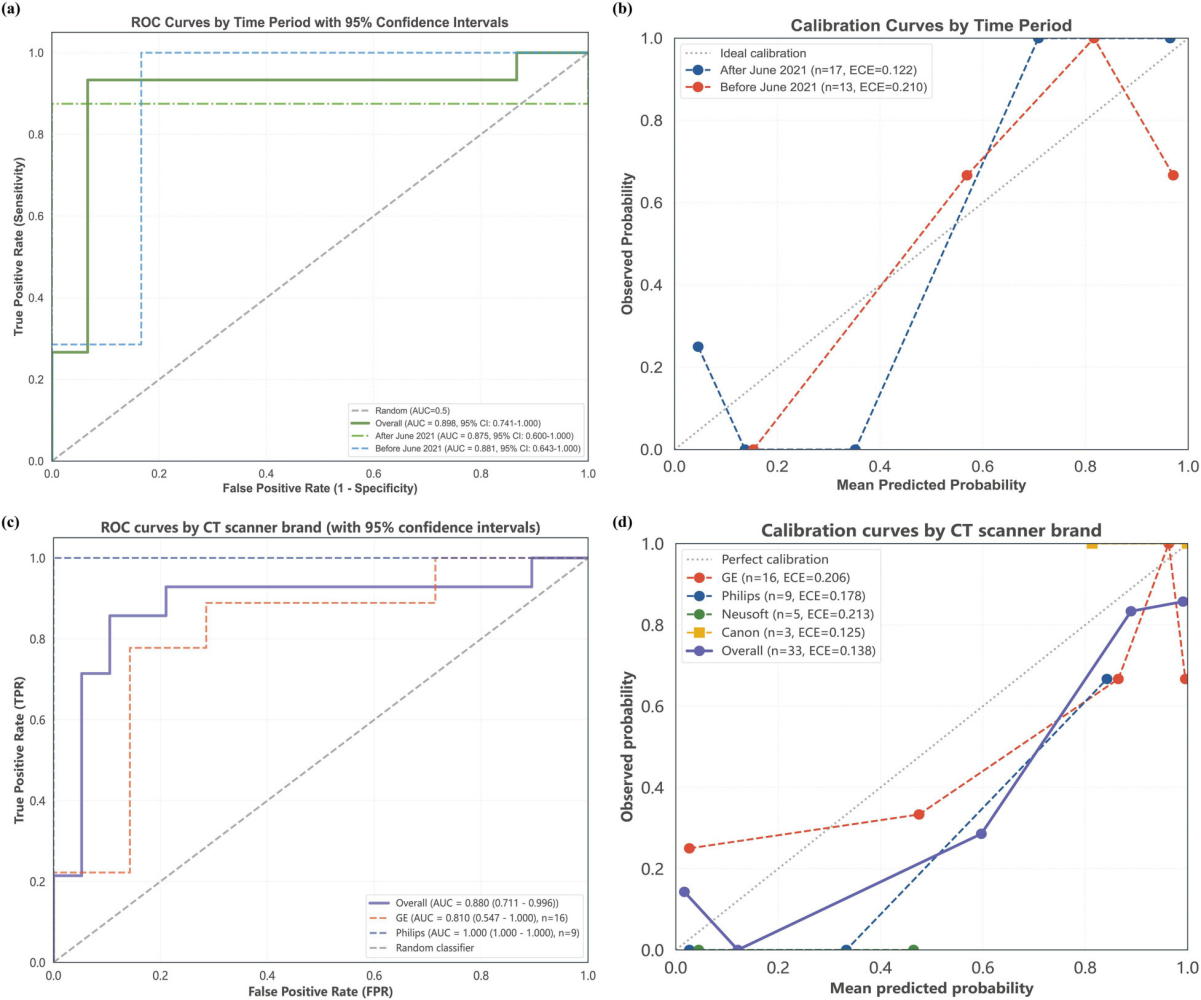
Robustness analysis against domain shifts. The model's performance under temporal drift (development test set) and across four CT scanner vendors (internal temporal test set) was evaluated. **(a)** ROC curves for temporal subgroups; **(b)** calibration curves for the temporal subgroups; **(c)** ROC curves across four CT scanner vendors; **(d)** calibration curves across vendors. CT, computed tomography; ROC, receiver operating characteristic.

To assess robustness to potential domain shifts, we performed subgroup analyses. Performance remained consistent across time periods when the development test set was split at its median date (Table 11). Similarly, across CT scanner vendors in the internal temporal validation cohort, diagnostic performance remained stable for the major manufacturers (Table 12). Calibration remained acceptable in all subgroups. These internal assessments indicate that the model is robust to temporal drift and variation across the primary CT platforms used in this study, as shown in Figure 8.

**Table 11:** Temporal domain shift analysis of the model on the development test set.

Time Period	Cases	Positive Cases	AUC (95% CI)	Sensitivity	Specificity	ECE	Brier
Overall	30	15	0.898 (0.746-1.000)	0.933	0.933	0.196	0.115
After June 2021	17	8	0.875 (0.571-1.000)	0.875	1	0.183	0.104
Before June 2021	13	7	0.881 (0.600-1.000)	1	0.833	0.236	0.130

AUC, area under the curve; ECE, expected calibration error.

**Table 12:** Model performance across different CT scanner manufacturers on the internal temporal test set.

CT Scanner Brand	Cases	Positive Cases	AUC (95% CI)	Sensitivity	Specificity	ECE	Brier Score
Overall	33	14	0.880 (0.718-0.996)	0.929	0.737	0.160	0.142
Canon	3	3	nan (nan-nan)	N/A	N/A	0.125	0.043
GE	16	9	0.810 (0.533-1.000)	0.889	0.714	0.206	0.189
Neusoft	5	0	nan (nan-nan)	N/A	N/A	0.213	0.110
Philips	9	2	1.000 (1.000-1.000)	1	0.714	0.219	0.110

CT, computed tomography; AUC, area under the curve; ECE, expected calibration error.

### Model interpretability and visualization

**Figure 9:**
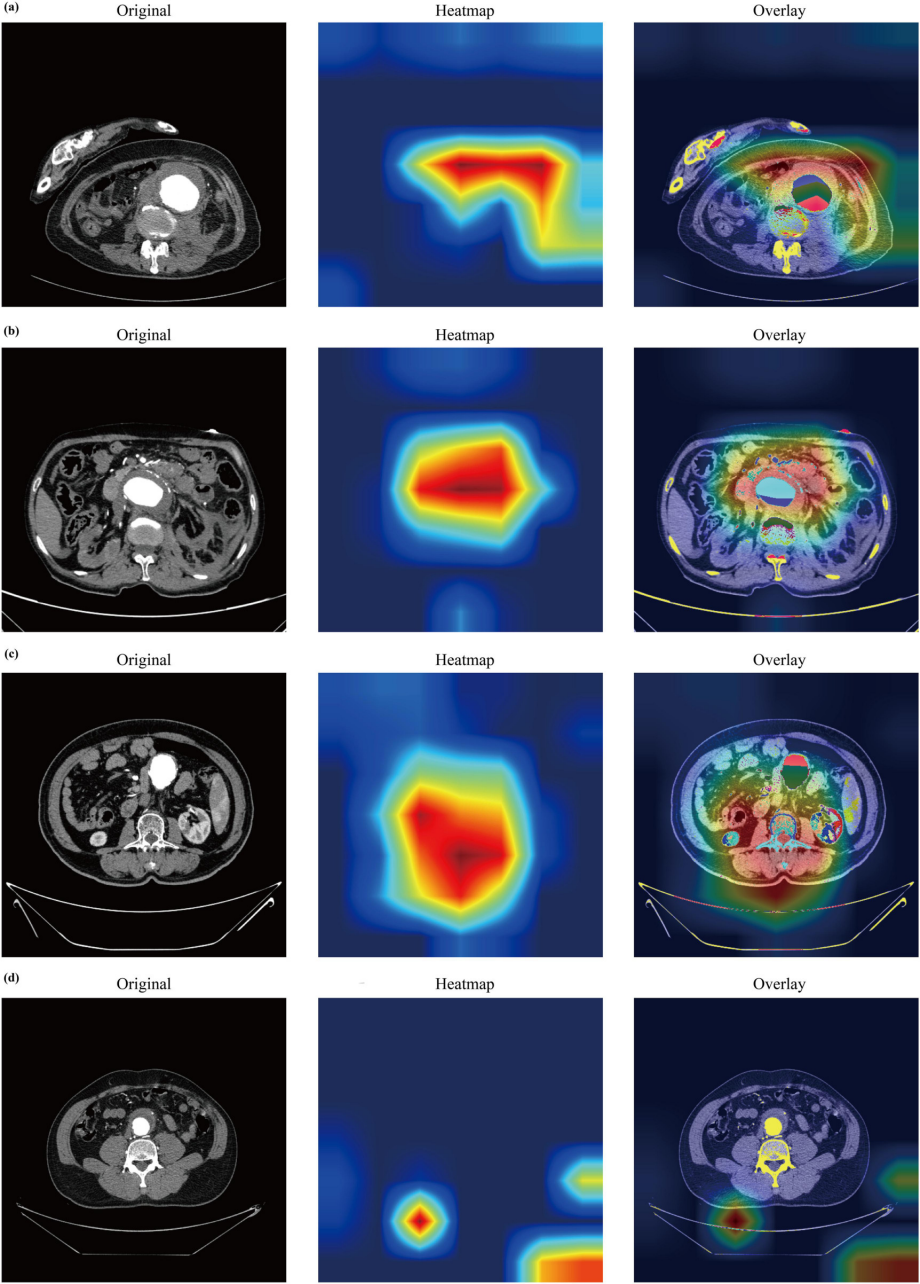
Grad-CAM visualizations of representative AAA cases from the internal temporal validation cohort. Heatmaps indicate model attention for predicting impending rupture (red = highest, blue = lowest). **(a)** True positive: The model focuses on the pathological aortic wall exhibiting high-risk CTA signs in a patient with impending rupture. **(b)** True negative: Minimal attention to the aortic wall in a stable patient correctly classified as low risk. **(c)** False positive: Attention directed to the peri-aneurysmal area in the absence of definitive high-risk CTA signs, leading to incorrect high-risk classification in a stable patient. **(d)** False negative: Attention shifts to anatomically irrelevant structures, failing to capture the diseased aortic segment. Grad-CAM, Gradient-Weighted Class Activation Mapping; CTA, computed tomography angiography; AAA, abdominal aortic aneurysm.

Grad-CAM heatmaps highlighted the regions most influential to model predictions (Figure 9). In the independent temporal validation cohort (*n* = 33), two senior vascular radiologists, blinded to model outputs and final diagnoses, independently assessed plausibility for all 33 cases. The model's attention was anatomically plausible in 26 cases (78.8%), moderately plausible in 5 (15.2%), and implausible in 2 (6.1%), with moderate interrater agreement (Cohen's *κ* = 0.60). The single false-positive case showed partially shifted yet peri-aneurysmal attention (rated moderately plausible); conversely, a false-negative case exhibited decisive activation on irrelevant adjacent structures (rated implausible), visually explaining the error. These findings indicate that the model generally focuses on clinically relevant anatomy; occasional misplacements visible in heatmaps help explain specific errors and could trigger human-in-the-loop review. For the clinical modality, interpretability derived from feature importance analysis during selection (Figure 4a). The six selected features—WBC count, DBP, total cholesterol, creatinine, fibrinogen, and RBG—are intrinsically interpretable biomarkers with established physiological relevance to AAA pathophysiology. Heatmap generation required 6.45–7.46 s per case (median 7.02 s; see Supplementary Table S15).

## Discussion

The management of symptomatic, hemodynamically stable AAAs presents a time-sensitive challenge in emergency care. Existing deep-learning models predict long-term rupture risk in asymptomatic surveillance cohorts (26–28), but are designed for elective outpatient settings, rendering them unsuitable for acute triage requiring decisions within hours. In this acute context, clinicians must integrate imaging findings with the patient's systemic physiological state—a process unsupported by current AI tools, representing a notable gap in vascular AI applications (29). To address this unmet need, we developed and temporally validated an interpretable multimodal deep-learning framework tailored for emergency decision-making. This clinically grounded approach enables rapid rupture risk stratification by integrating sequential CTA morphology with pre-intervention biomarkers (inflammation, hemodynamic stress, metabolic state) via a BCA mechanism that mirrors clinical reasoning, with embedded Grad-CAM explainability providing anatomical plausibility checks and fostering clinical trust.

Our model prioritizes clinical utility over architectural novelty, processing standard axial CTA slices with routine admission biomarkers to align with emergency workflows while avoiding 3D reconstructions or expert-dependent feature engineering. Central to this design, the BCA mechanism enables iterative interaction between imaging and clinical data, unlike late-stage concatenation or unidirectional attention in prior multimodal approaches (5, 28). This allows clinical context (e.g., elevated WBC, low blood pressure) to guide attention to relevant imaging regions while infusing imaging features with clinical interpretation. Ablation studies confirmed this approach outperforms simpler fusion strategies by capturing synergistic signals, such as systemic inflammation exacerbating local wall stress, that herald impending rupture (Table 5). We propose that this mimics expert clinical reasoning, wherein imaging findings and physiological status are interpreted together rather than in isolation.

To further ensure robustness, the model was developed within a propensity-score-matched cohort. Patients were matched on age, sex, and maximum aneurysm diameter—variables selected *a priori* as the most clinically established confounders of rupture risk. The maximum diameter is the primary determinant of rupture risk and a key trigger for elective repair; sex is critical as women exhibit higher rupture risk at smaller diameters; and age is an essential demographic confounder in AAA outcomes (15). This matching design ensures that predictive signals extend beyond these traditional risk factors. Additionally, internal temporal validation on a small but independent cohort (*n* = 33) provided preliminary evidence of temporal stability (AUC 0.880), serving as a foundation for future external validation.

Building on these advantages, our work addresses a complementary clinical gap in the AAA AI landscape. While prior research focused on long-term rupture risk in asymptomatic patients using geometric or fluid-dynamic analyses for elective management (28), our model targets a different scenario: predicting impending rupture within hours in symptomatic yet stable patients. This study demonstrates AI feasibility for acute AAA presentation through a methodological shift from precision simulation to rapid data fusion. Complementing long-term tools, it addresses an underserved acute phase, enabling future integration with surveillance models toward a continuum of AI-assisted care.

Clinical plausibility is supported by a transparent feature selection process balancing discriminative power and robustness via the composite score. The six selected biomarkers achieved the highest composite score among all candidate sets (Supplementary Table S13), reflecting an optimal trade-off between performance and generalizability. This panel aligns with impending rupture pathophysiology: systemic inflammation (elevated WBC), hemodynamic stress (low DBP), metabolic dysregulation (elevated RBG), and a prothrombotic state (elevated fibrinogen) (30, 31). Despite moderate bootstrap stability (55.55%), this subset outperformed less stable alternatives. We hypothesize that BCA integrates these biomarkers with imaging, mirroring clinical reasoning—supported by sensitivity analysis confirming robustness against treatment confounding using pre-intervention data.

The model's performance reflects a deliberate trade-off tailored to symptomatic AAA triage. By prioritizing high sensitivity and NPV, it reduces missed rupture risk from ∼62% to 12.5%, at the cost of moderate specificity and some overestimation. This safety-first approach suits life-threatening emergencies, where false negatives (delayed intervention, fatal rupture) far outweigh false positives.

While encouraging, these metrics must be interpreted in light of a key consideration: the inherent circularity introduced by an endpoint definition that incorporates established radiological signs of rupture. Rather than diminishing clinical utility, this circularity reframes the clinical problem: the true challenge lies not in the absence of these signs, but in their subjective interpretation across practitioners. To address this directly, we performed a sensitivity analysis restricted to cases with hard objective evidence (contained rupture or intraoperative confirmation), thereby excluding those defined solely by high-risk CTA signs. In this more rigorously defined subset, the model maintained robust performance, with AUC remaining at 0.75 in both cohorts and high sensitivity and negative predictive value, metrics critical for safe emergency rule-out. The expected decline in AUC relative to the full cohort (from 0.898) reflects the smaller sample size, lower event prevalence, and inherent diagnostic difficulty of this subgroup, rather than a failure of model generalizability. These findings thus contextualize and substantiate the model's utility in detecting subtle high-risk signs where clinical uncertainty is greatest.

Missing data analysis revealed that patients with complete pre-intervention data were higher-risk, with a greater prevalence of hypertension, alcohol use, and impending rupture, a pattern consistent with real-world practice where more symptomatic patients undergo comprehensive testing. Despite this more challenging cohort, the model sustained strong performance (AUC 0.864 and 0.857), affirming its robustness precisely where risk stratification matters most.

Grad-CAM visualizations achieved 78.8% anatomical plausibility per blinded clinician assessment, supporting face validity and fostering clinical trust. Importantly, this method also revealed a potential failure mode: in one critical false-negative case, attention shifted to irrelevant anatomy, a pattern suggestive of spurious correlations, though this interpretation remains speculative given the single observation (32). Nevertheless, this visual cue serves as a practical trigger for human-in-the-loop review, flagging predictions with implausible heatmaps for audit. These findings warrant cautious interpretation: Grad-CAM highlights statistically correlated regions but does not establish causality or specific mechanisms. The principal value of explainability here lies in enhancing transparency, enabling sanity checks, and facilitating human-AI collaboration. We acknowledge, however, that heatmaps show where the model “looked” without explaining biological significance—a direct pathophysiological link that remains elusive and requires future histopathological or biomechanical correlation.

Balancing the pre-trained models’ utility with CT semantics preservation was key in our pipeline. Converting windowed arterial-phase CTA to ImageNet-normalized RGB format outperformed a CT-native single-channel approach, despite obscuring absolute HU values. For rapid rupture classification, transfer learning benefits outweigh densitometric precision. This trade-off is clinically sound: standard arterial windowing maintains radiological contrast, while ImageNet normalization enables detection of subtle morphological signs via pre-learned features. Our approach prioritizes high-level feature transfer, acknowledging its limitations for tasks requiring HU thresholding. Future domain-adaptive pre-training may harmonize both paradigms.

Conventional practice for identifying impending AAA rupture relies on subjective, experience-dependent interpretation of CTA signs and clinical heuristics (2, 33), lacking the precision and safety required for emergency triage. Recent studies proposing high-attenuation CT values as predictors remain unadopted (34). Given the limitations of current approaches, we developed a multimodal BCA-based model integrating imaging with acute physiology. Although its higher AUC did not reach statistical significance versus baselines—likely due to limited sample size—the model excelled on emergency-relevant metrics: high NPV for safe rule-out, superior calibration, and greater net clinical benefit across risk thresholds in DCA. By prioritizing clinical utility over statistical discrimination, our model offers a unified, data-driven assessment that enhances both safety and efficiency in symptomatic AAA triage.

This proof-of-concept, single-center study validates a multimodal risk-stratification framework, rather than a deployable tool. Prospective multicenter validation is needed to confirm efficacy and quantify the impact on clinical outcomes. Our internal temporal validation provides a methodological blueprint for such evaluation. The extensible BCA architecture could later incorporate dynamic data streams to further enrich multimodal assessment. This work lays the groundwork for AI decision-support systems that integrate multimodal data into acute care, augmenting clinical expertise in time-sensitive vascular emergencies.

## Limitations

This study has several limitations. First, the retrospective single-center design and modest temporal validation cohort limit generalizability, warranting prospective multicenter external validation as a critical next step. Second, the composite endpoint inherently incorporates radiological signs, introducing potential circularity; however, sensitivity analyses confirmed that predictions are not driven solely by this definition. Third, calibration shows slight overestimation at the highest risk levels. This safety-cautious bias aligned with emergency triage priorities, though recalibration is needed for precise individual estimation. Fourth, manual scan boundary initialization, while required, imposes a modest workflow burden that future automation could eliminate. Finally, although Grad-CAM does not establish causality, its anatomical plausibility supports clinical trust and oversight, positioning this as an interpretable tool rather than a black-box system.

## Conclusion

We developed an interpretable multimodal deep learning framework integrating CTA with clinical biomarkers for rapid stratification of impending AAA rupture in symptomatic patients. The model demonstrated robust performance in temporal validation, addressing a critical gap in emergency care. While prospective validation remains essential, this work provides a clinically grounded, explainable foundation for AI-assisted decisions in time-sensitive vascular emergencies.

## Data Availability

The raw data supporting the conclusions of this article will be made available by the authors, without undue reservation.
