# Supplementary material for: A multimodal deep learning model for predicting impending rupture in symptomatic abdominal aortic aneurysms using CTA and clinical data
Source: Front Cardiovasc Med. 2026 Apr 7;13:1771669. doi: 10.3389/fcvm.2026.1771669 (PMC13095579; doi:10.3389/fcvm.2026.1771669)
Supplement: Supplementary file 1 [file Datasheet1.docx]

Figure S1. Six clinical features selected through logistic regression.


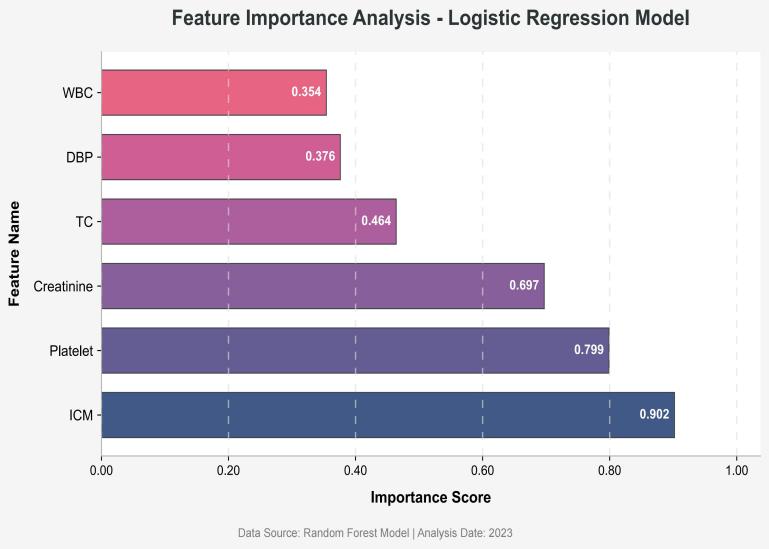


Figure S2. Six clinical features selected through extreme gradient boosting.


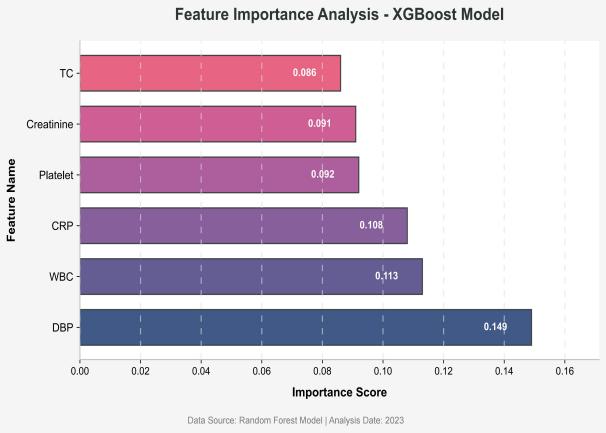


Figure S3. Six clinical features selected through minimum redundancy maximum relevance.


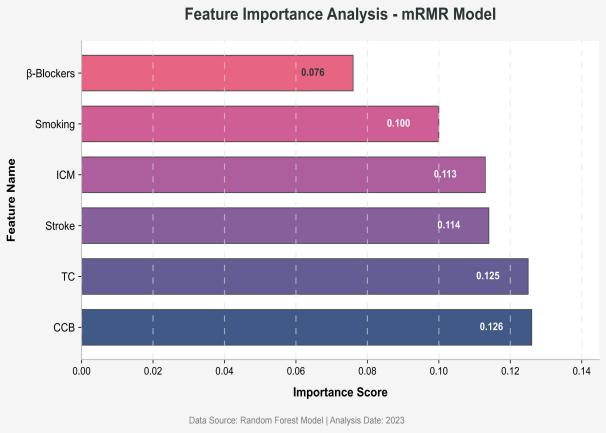
Figure S4. Six clinical features selected through gradient-boosted decision trees.


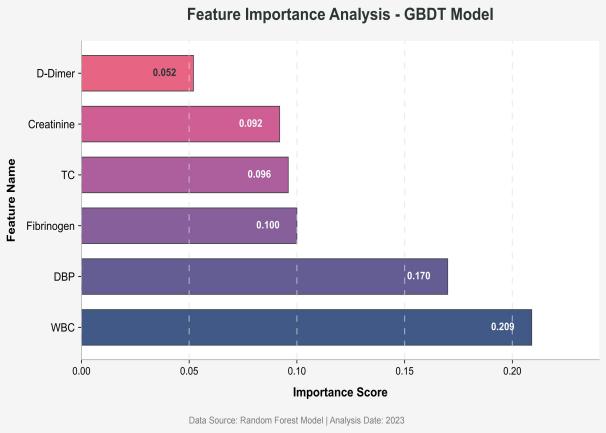


Figure S5. The performance of the feature fusion model based on AlexNet. (a) ROC curve of the multimodal model; (b) Confusion matrices of the multimodal model; (c) Calibration curve; (d) Decision curve analysis; (e) Bootstrap confidence intervals. ROC: receiver operating characteristic; AUC: Area under the curve.


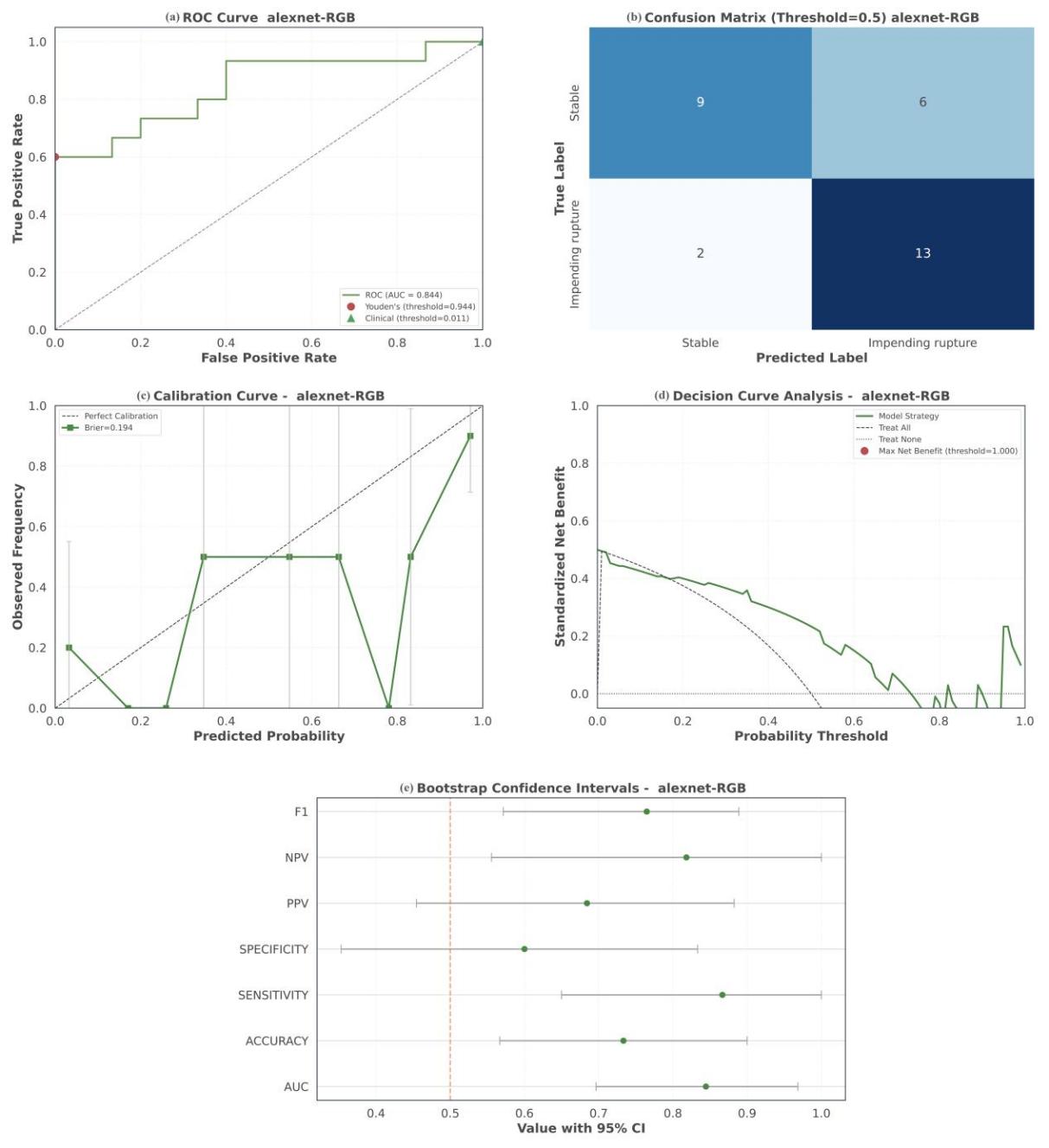


Figure S6. The performance of the feature fusion model based on ResNet18. (a) ROC curve of the multimodal model; (b) Confusion matrices of the multimodal model; (c) Calibration curve; (d) Decision curve analysis; (e) Bootstrap confidence intervals. ROC: receiver operating characteristic; AUC: Area under the curve.


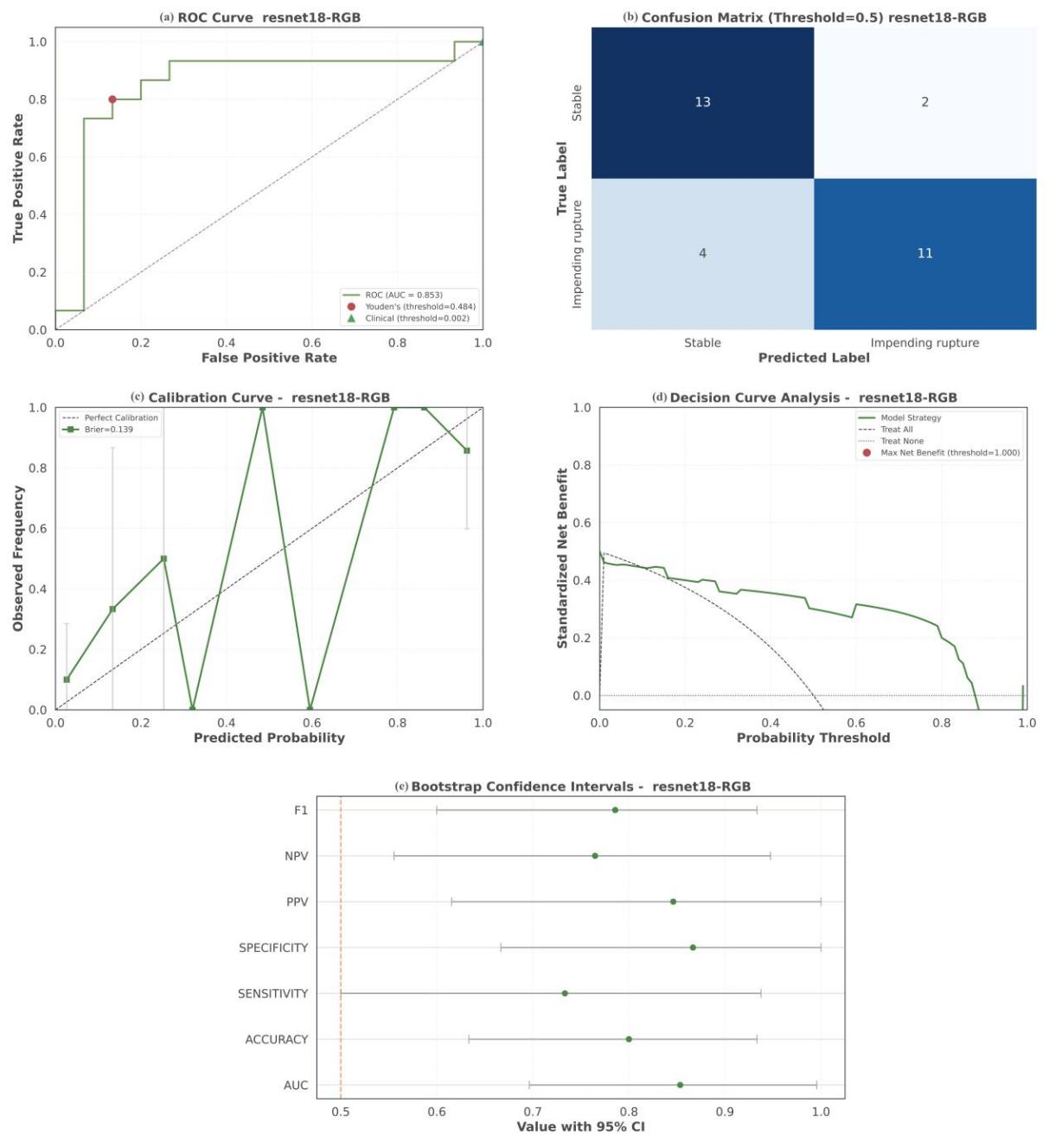


Figure S7. The performance of the feature fusion model based on ResNet101. (a) ROC curve of the multimodal model; (b) Confusion matrices of the multimodal model; (c) Calibration curve; (d) Decision curve analysis; (e) Bootstrap confidence intervals. ROC: receiver operating characteristic; AUC: Area under the curve.





Figure S8. The performance of the feature fusion model based on DenseNet121. (a) ROC curve of the multimodal model; (b) Confusion matrices of the multimodal model; (c) Calibration curve; (d) Decision curve analysis; (e) Bootstrap confidence intervals. ROC: receiver operating characteristic; AUC: Area under the curve.


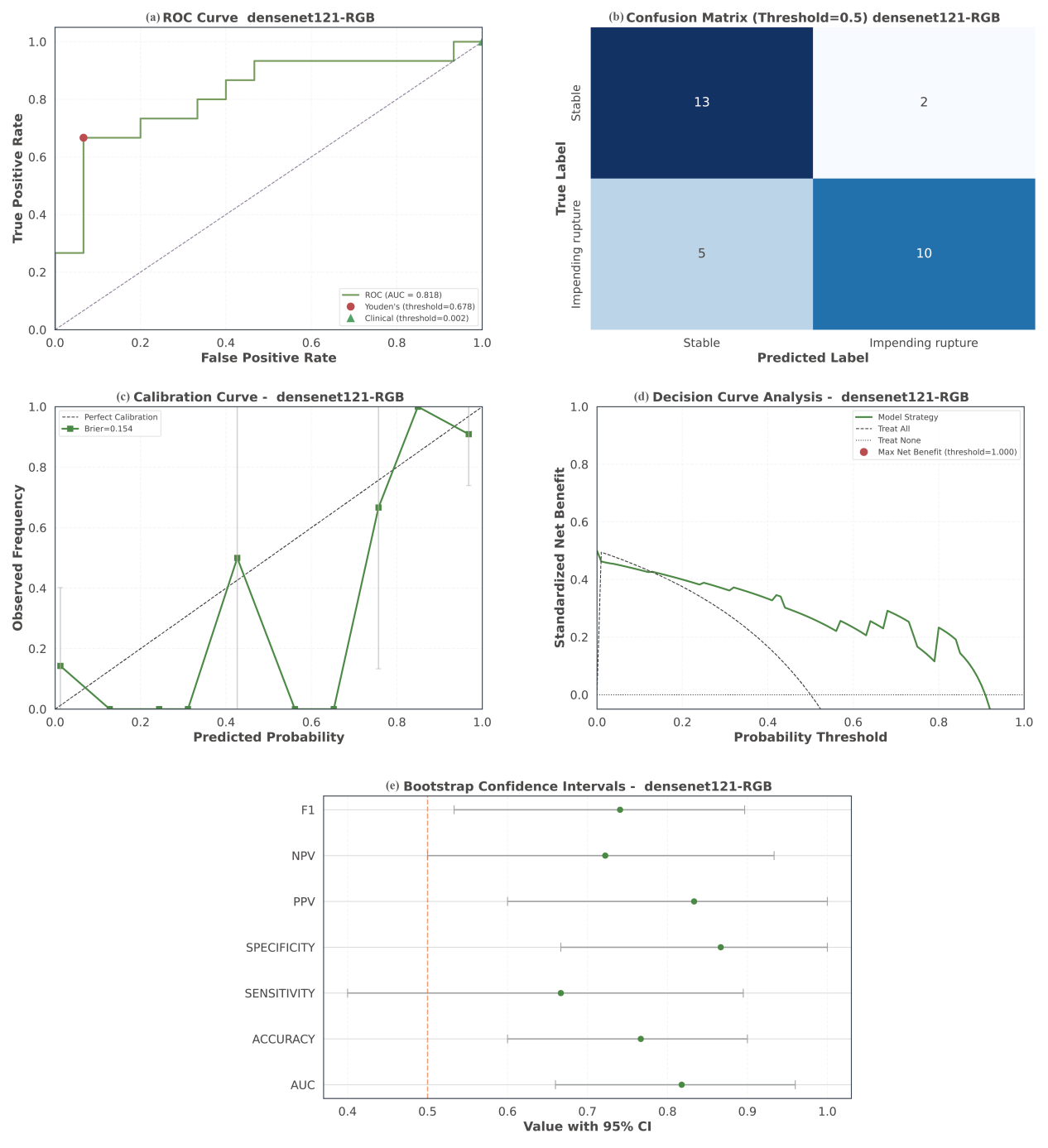


Figure S9. The performance of the feature fusion model based on VGG16. (a) ROC curve of the multimodal model; (b) Confusion matrices of the multimodal model; (c) Calibration curve; (d) Decision curve analysis; (e) Bootstrap confidence intervals. ROC: receiver operating characteristic; AUC: Area under the curve.

**
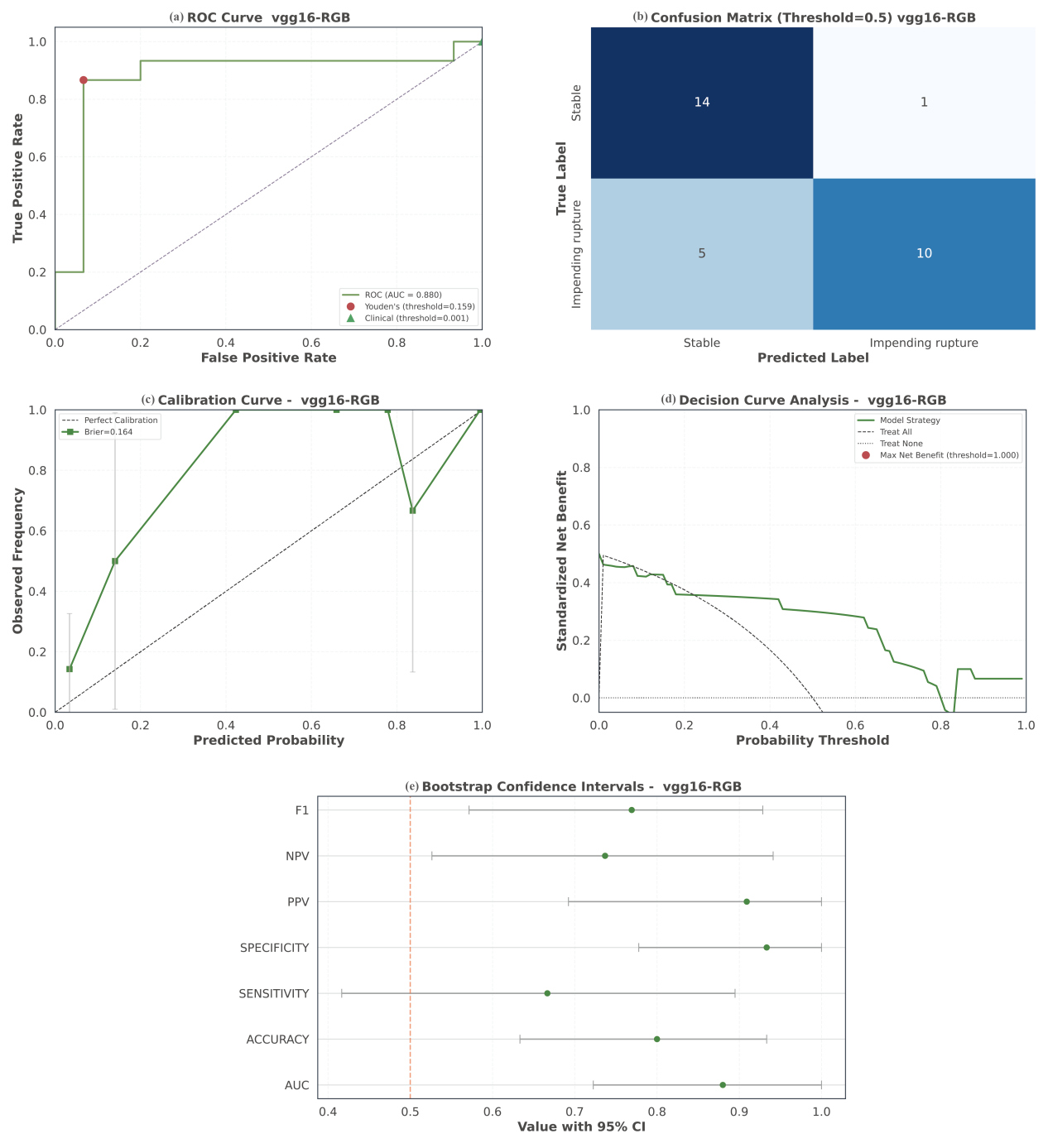
**

Figure S10. The performance of the feature fusion model based on ViT-B/16. (a) ROC curve of the multimodal model; (b) Confusion matrices of the multimodal model; (c) Calibration curve; (d) Decision curve analysis; (e) Bootstrap confidence intervals. ROC: receiver operating characteristic; AUC: Area under the curve.


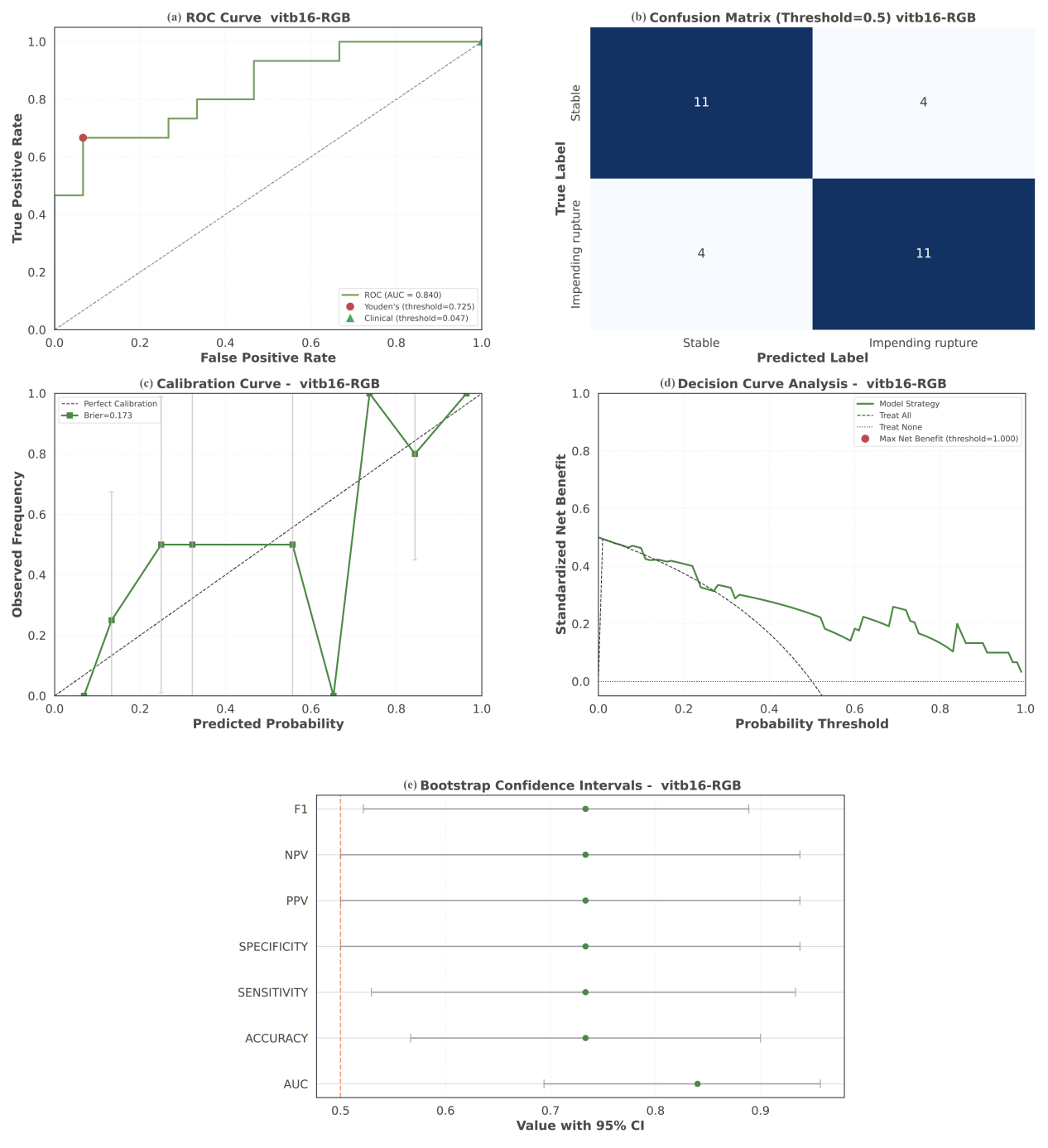


Figure S11. The performance of the feature fusion model based on MedViT. (a) ROC curve of the multimodal model; (b) Confusion matrices of the multimodal model; (c) Calibration curve; (d) Decision curve analysis; (e) Bootstrap confidence intervals. ROC: receiver operating characteristic; AUC: Area under the curve.


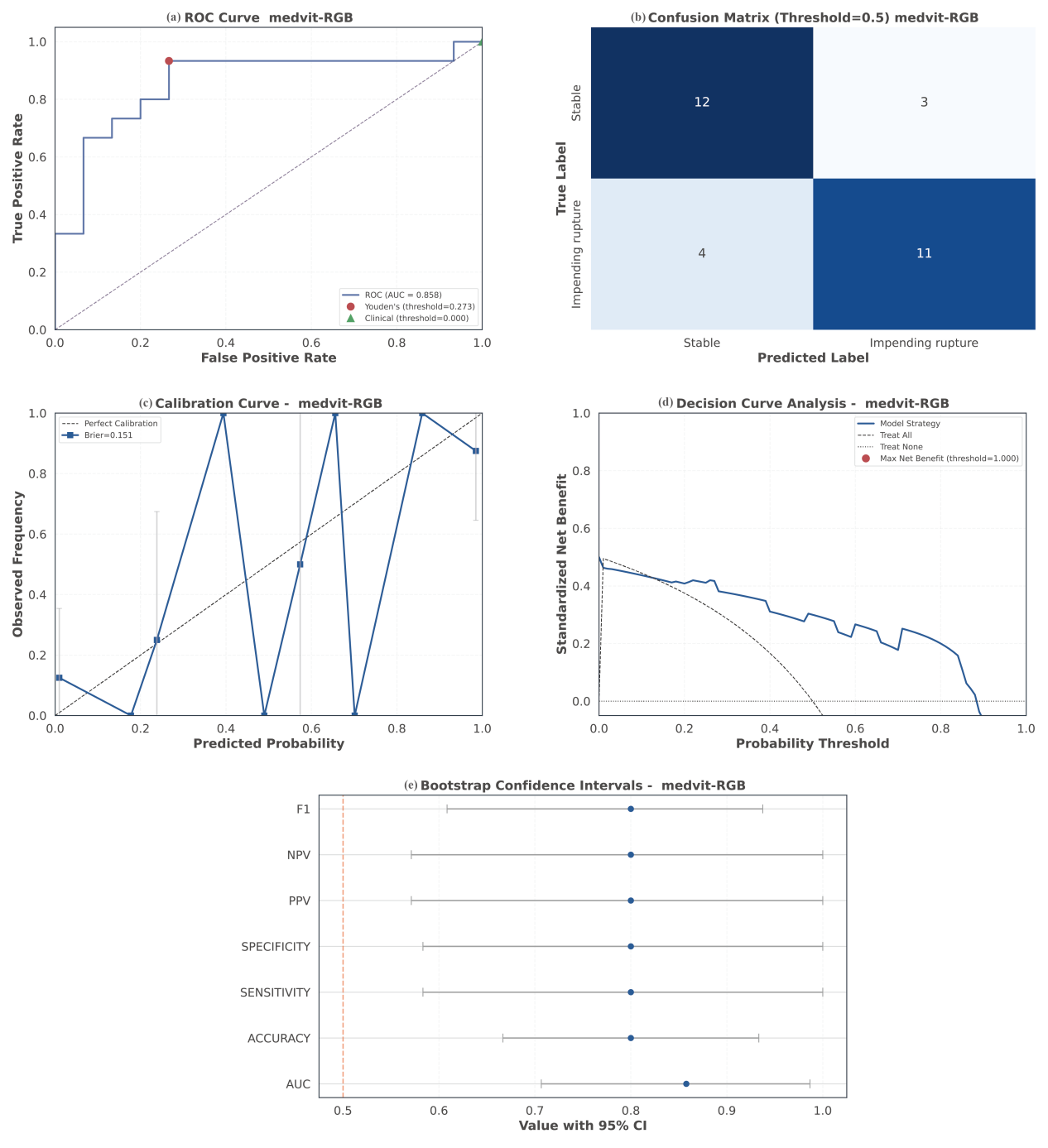


Figure S12. Training and validation losses based on the ResNet50 backbone. **
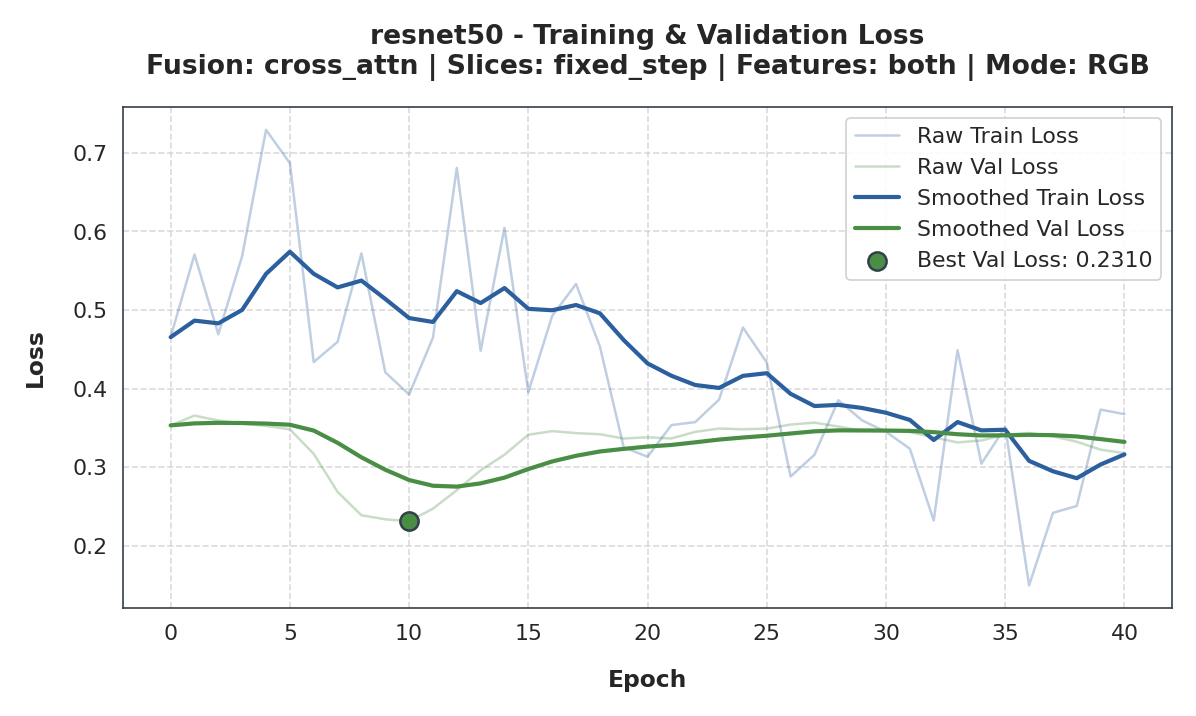
**

Figure S13. The performance of the feature fusion model based on ResNet50 in the grayscale mode of the development cohort. (a) ROC curve of the multimodal model; (b) Confusion matrices of the multimodal model; (c) Calibration curve; (d) Decision curve analysis; (e) Bootstrap confidence intervals. ROC: receiver operating characteristic; AUC: Area under the curve.


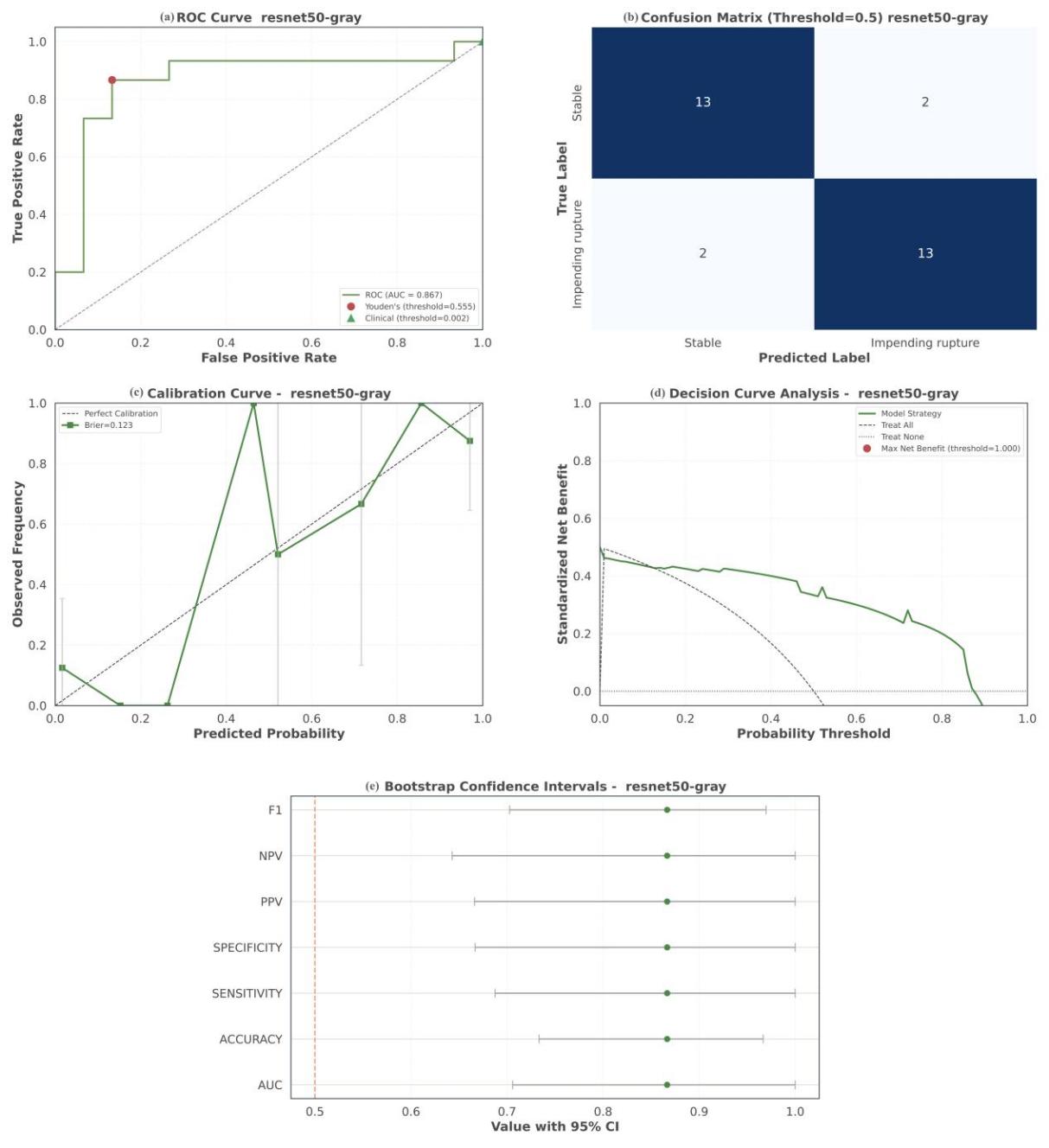


Figure S14. The performance of the feature fusion model based on ResNet50 in the grayscale mode of the internal temporal validation cohort. (a) ROC curve of the multimodal model; (b) Confusion matrices of the multimodal model; (c) Calibration curve; (d) Decision curve analysis; (e) Bootstrap confidence intervals. ROC: receiver operating characteristic; AUC: Area under the curve.


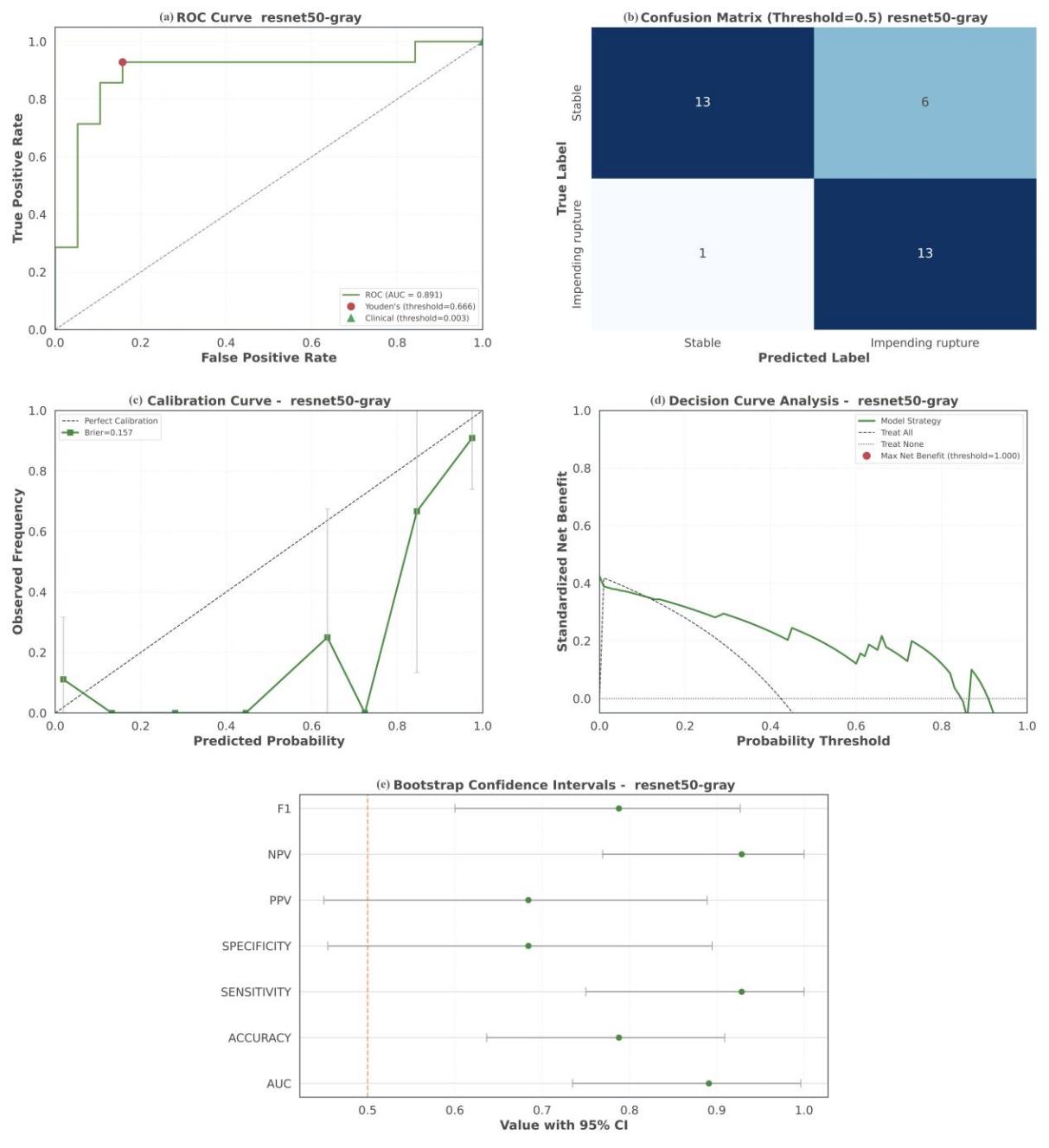


Figure S15. ROC and calibration curves for evidence-based subsets in the development test cohort. Panels show ROC curves (left) and calibration curves (right) for: (a, b) overall; (c, d) imaging‑objective; (e, f) hard‑objective; (g, h) contained rupture on CTA only; (i, j) high‑risk CTA signs only. Brier scores are displayed in each calibration plot.


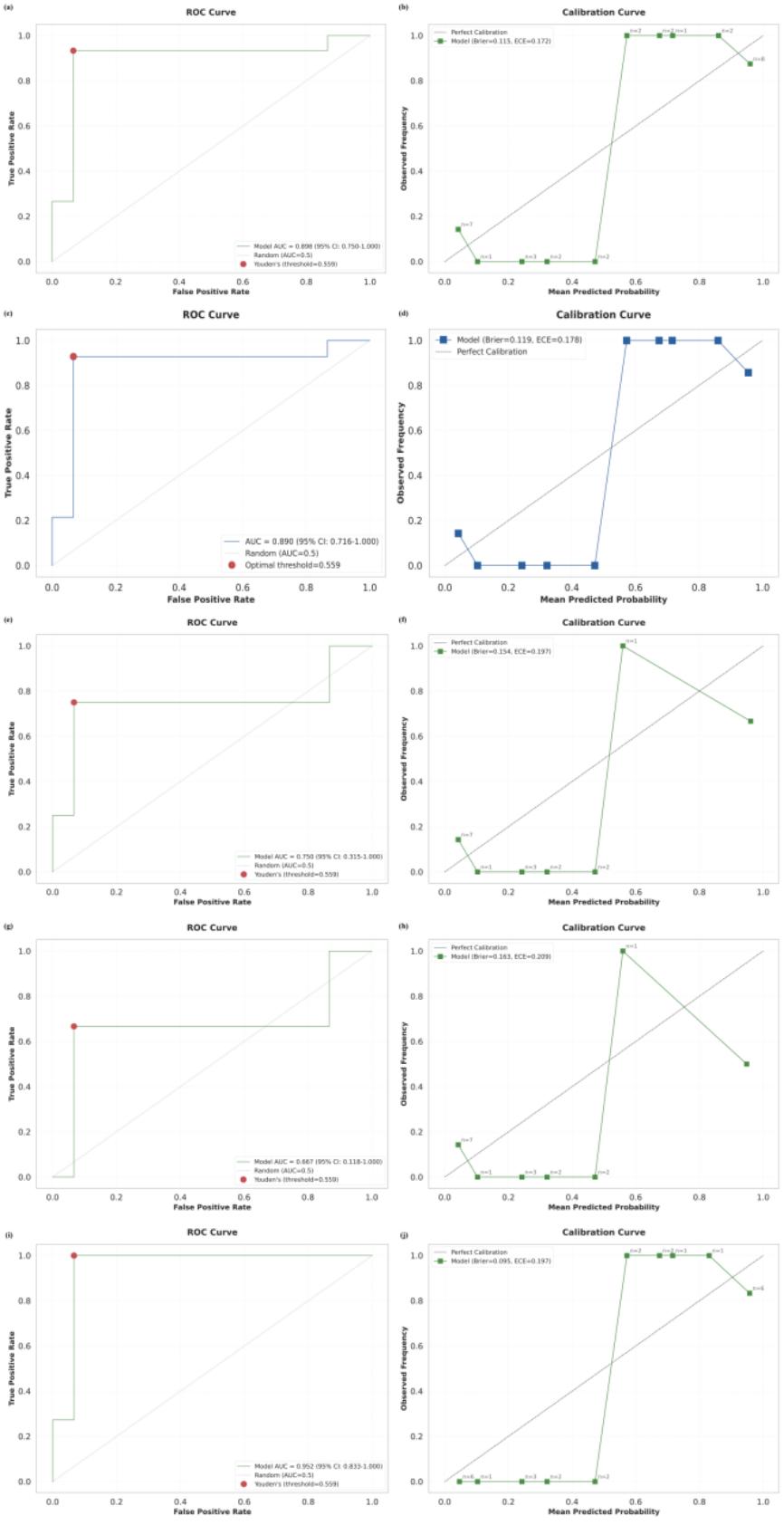


Figure S16. ROC and calibration curves for evidence-based subsets in the internal temporal validation cohorts. Panels show ROC curves (left) and calibration curves (right) for: (a, b) overall; (c, d) imaging-objective; (e, f) hard-objective; (g, h) contained rupture on CTA only; (i, j) high-risk CTA signs only. Brier scores are displayed in each calibration plot.


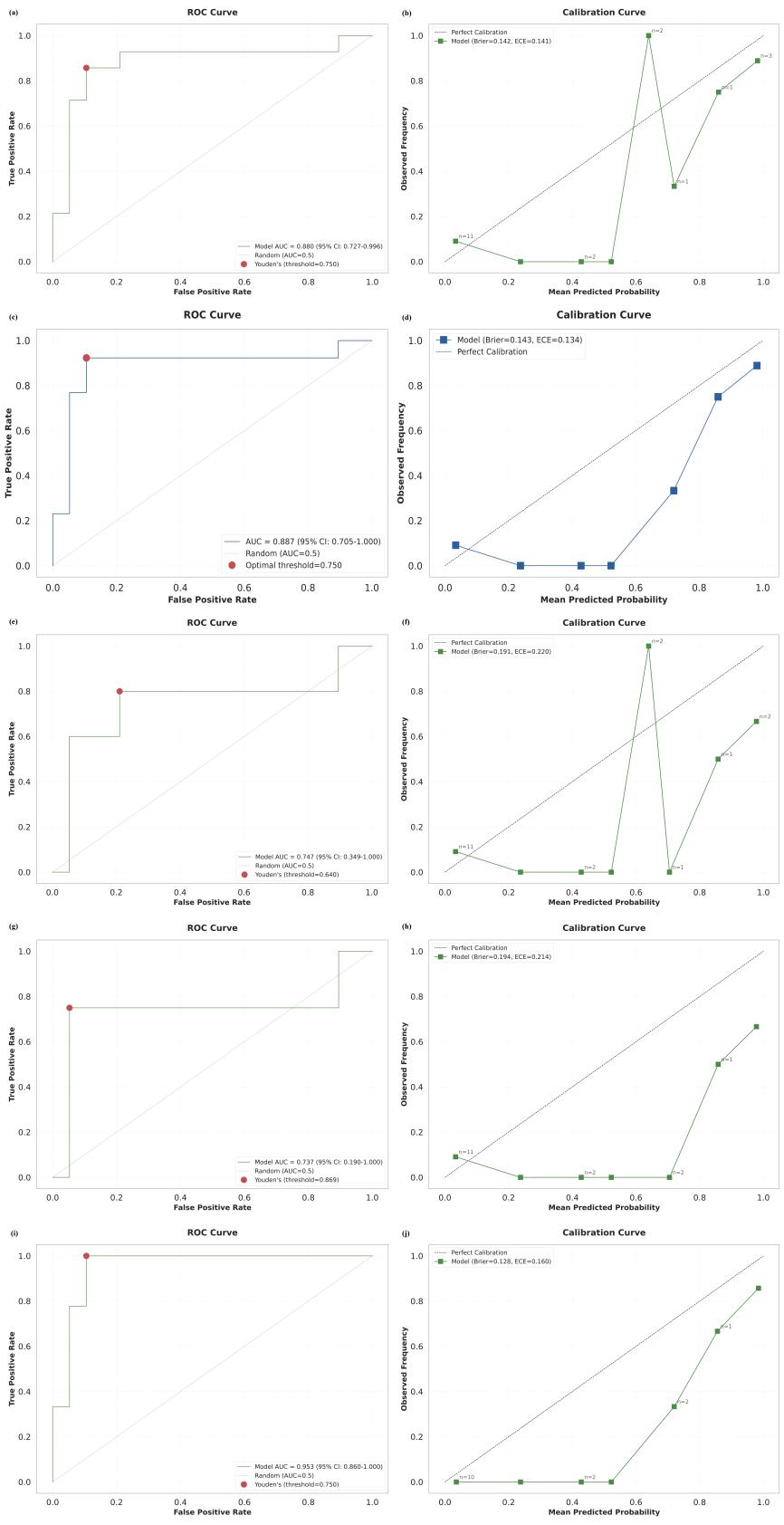


Figure S17. Sensitivity analysis of the multimodal model on the strictly pre-intervention subsets. (a) ROC curve on the development test set; (b) Calibration curve on the development test set; (c) Decision curve analysis on the development test set.(d) ROC curve on the internal temporal validation set; (e) Calibration curve on the internal temporal validation set; (f) Decision curve analysis on the internal temporal validation set. ROC: receiver operating characteristic.


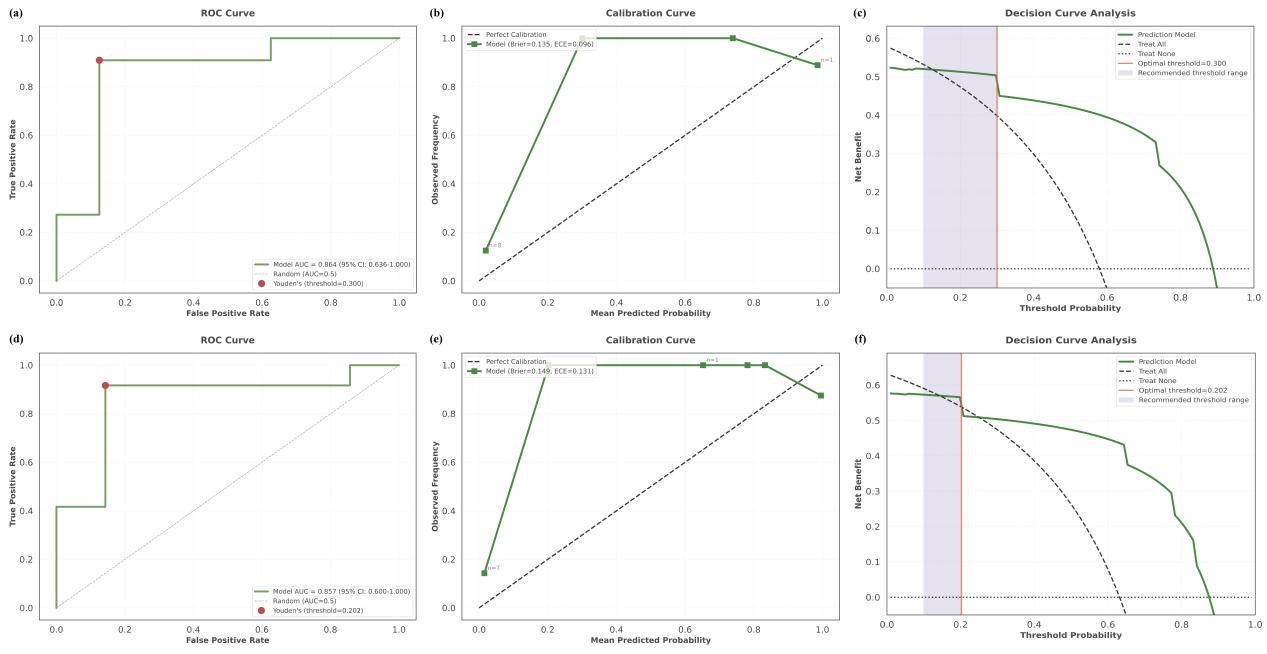


**Definition**

The AAA is defined as the diameter of the abdominal aorta at least 3 cm, and the maximum diameter was measured by two radiologists based on multiplanar reconstruction.

Smoking history was defined as an individual's lifetime consumption of 100 or more cigarettes. Drinking history was defined as daily consumption of at least 50 mL of liquor, at least once a week, for a duration of at least 6 months, including current drinkers and those who stopped drinking but met the aforementioned criteria.

Definition of baseline variables: systolic blood pressure and diastolic blood pressure were measured at the first admission.

The patient's coronary heart disease history, diabetes, hypertension, and stroke history were obtained according to the conversation and inquiry between the doctor in charge and the patient and his or her family, as well as previous examination reports or previous medical records provided by the patient and his or her family.

Physical and chemical examination: low-density lipoprotein cholesterol (LDL-C), blood glucose, hemoglobin, white blood cells, platelets were recorded for the first time after admission.

Prior medication was recorded according to electronic medical records.

Formula.

1.
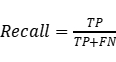


1.
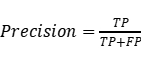


1.
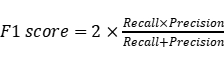


1.
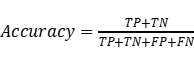


TN: True Negative; FP: False Positive; FN: False Negative; TP: True Positive.
