# Supplementary material for: A multimodal deep learning model for predicting impending rupture in symptomatic abdominal aortic aneurysms using CTA and clinical data
Source: Front Cardiovasc Med. 2026 Apr 7;13:1771669. doi: 10.3389/fcvm.2026.1771669 (PMC13095579; doi:10.3389/fcvm.2026.1771669)
Supplement: Supplementary file 2 [file Datasheet2.docx]

Table S1. Key objective timestamps and initial clinical characteristics for the development cohort.

| Patient_id | CTA Date | Scan Time | Blood Draw Date | Phlebotomy Time | Imaging Device | Intervention Date | Intervention Time | Death Date | Death Time |
| --- | --- | --- | --- | --- | --- | --- | --- | --- | --- |
| 15 | 2017/3/16 | 9:32 | 2017/3/16 | 10:25 | Neusoft | 2017/3/24 | 14:26 |  |  |
| 31 | 2017/12/4 | 15:43 | 2017/12/4 | 15:19 | Neusoft | 2017/12/15 | 14:29 |  |  |
| 38 | 2017/8/20 | 9:54:00 | 2017/8/20 |  | Neusoft | 2017/9/1 | 9:08 |  |  |
| 47 | 2017/11/19 | 17:59 | 2017/11/19 |  | Neusoft | 2017/11/24 | 13:56 |  |  |
| 52 | 2017/6/11 | 13:15 | 2017/6/11 | 7:07 | Neusoft | 2017/6/11 | 22:08 |  |  |
| 80 | 2017/12/18 | 13:37 | 2017/12/18 | 6:39 | Neusoft | 2018/1/7 | 2:48 |  |  |
| 84 | 2017/12/20 | 11:04 | 2017/12/20 | 11:29 | Neusoft | 2018/1/8 | 10:23 |  |  |
| 89 | 2018/2/24 | 16:33 | 2018/2/24 | 15:29 | Neusoft |  |  |  | 22:09 |
| 91 | 2018/3/6 | 14:43 | 2018/3/6 | 15:34 | Neusoft | 2018/3/19 | 1:56 |  |  |
| 101 | 2018/3/27 | 1:34 | 2018/3/27 | 8:14 | Neusoft | 2018/3/27 | 14:04 |  |  |
| 115 | 2018/5/20 | 1:58 | 2018/5/20 | 1:51 | Neusoft | 2018/6/6 | 5:08 |  |  |
| 120 | 2018/5/22 | 10:38 | 2018/5/22 |  | Neusoft | 2018/6/6 | 15:42 |  |  |
| 127 | 2018/6/10 | 2:19 | 2018/6/10 | 1:33 | Neusoft | 2018/6/10 | 4:57 |  |  |
| 133 | 2018/6/28 | 21:46 | 2018/6/28 | 21:33 | Neusoft | 2018/6/29 | 8:32 |  |  |
| 143 | 2018/7/14 | 13:31 | 2018/7/14 | 5:56 | Neusoft | 2018/7/31 | 23:06 |  |  |
| 146 | 2018/7/30 | 20:25 | 2018/7/30 | 2:56 | Neusoft | 2018/7/30 | 7:26 |  |  |
| 149 | 2018/9/15 | 10:48 | 2018/9/15 | 10:01 | Neusoft | 2018/9/21 | 22:40 |  |  |
| 155 | 2018/10/9 | 10:17 | 2018/10/9 |  | Neusoft | 2018/10/14 | 4:15 |  |  |
| 158 | 2018/10/22 | 9:15 | 2018/10/22 |  | Neusoft | 2018/10/22 | 15:48 |  |  |
| 162 | 2018/11/5 | 1:47 | 2018/11/5 | 16:09 | Neusoft | 2018/11/6 | 5:03 |  |  |
| 171 | 2018/11/20 | 8:51 | 2018/11/20 | 8:01 | Neusoft | 2018/11/29 | 18:02 |  |  |
| 173 | 2018/12/6 | 23:48 | 2018/12/6 |  | Neusoft | 2018/12/13 | 5:38 |  |  |
| 175 | 2018/12/21 | 8:02 | 2018/12/21 |  | Neusoft | 2019/1/9 | 7:20 |  |  |
| 177 | 2018/12/26 | 23:41 | 2018/12/26 | 22:35 | Neusoft | 2018/12/27 | 13:10 |  |  |
| 179 | 2018/12/30 | 13:08 | 2018/12/30 | 12:52 | Neusoft | 2018/12/30 | 19:18 |  |  |
| 183 | 2019/4/23 | 21:25 | 2019/4/23 | 20:49 | Neusoft | 2019/4/24 | 15:48 |  |  |
| 186 | 2019/5/15 | 15:22 | 2019/5/15 |  | Neusoft | 2019/6/1 | 3:08 |  |  |
| 187 | 2019/6/28 | 10:15 | 2019/6/28 | 9:46 | Neusoft | 2019/6/28 | 15:21 |  |  |
| 194 | 2019/6/28 | 18:38 | 2019/6/28 | 17:28 | Neusoft | 2019/7/14 | 8:45 |  |  |
| 196 | 2019/7/1 | 16:01 | 2019/7/1 | 16:59 | Neusoft | 2019/7/1 | 22:33 |  |  |
| 197 | 2019/7/6 | 18:55 | 2019/7/6 | 9:14 | Neusoft | 2019/7/6 | 15:21 |  |  |
| 204 | 2019/7/10 | 21:14 | 2019/7/10 | 20:55 | GE Healthcare | 2019/7/11 | 2:28 |  |  |
| 208 | 2019/8/12 | 11:29 | 2019/8/12 | 9:20 | Philips Healthcare | 2019/8/30 | 10:17 |  |  |
| 214 | 2019/8/13 | 8:09 | 2019/8/13 | 7:32 | GE Healthcare | 2019/8/13 | 19:49 |  |  |
| 215 | 2019/8/27 | 19:41 | 2019/8/27 | 5:26 | GE Healthcare | 2019/9/8 | 8:47 |  |  |
| 217 | 2019/9/21 | 4:42 | 2019/9/21 | 2:31 | GE Healthcare | 2019/9/27 | 18:04 |  |  |
| 221 | 2019/10/14 | 14:14 | 2019/10/14 | 5:45 | Philips Healthcare | 2019/10/19 | 12:18 |  |  |
| 225 | 2019/10/16 | 14:56 | 2019/10/16 | 14:19 | Philips Healthcare | 2019/10/16 | 19:03 |  |  |
| 232 | 2019/10/21 | 8:58 | 2019/10/21 | 12:43 | Philips Healthcare | 2019/10/22 | 8:16 |  |  |
| 239 | 2019/10/23 | 10:38 | 2019/10/23 | 9:55 | Philips Healthcare | 2019/11/4 | 7:17 |  |  |
| 242 | 2019/10/21 | 13:58 | 2019/10/21 | 6:07 | Philips Healthcare | 2019/11/4 | 0:31 |  |  |
| 252 | 2019/10/24 | 16:56 | 2019/10/24 | 22:55 | GE Healthcare | 2019/11/11 | 21:10 |  |  |
| 262 | 2019/11/5 | 10:13 | 2019/11/5 | 9:16 | Philips Healthcare | 2019/11/15 | 10:24 |  |  |
| 263 | 2019/11/7 | 15:16 | 2019/11/7 | 14:38 | Neusoft | 2019/11/7 | 19:07 |  |  |
| 266 | 2019/12/6 | 22:40 | 2019/12/6 | 22:13 | GE Healthcare | 2019/12/15 | 2:19 |  |  |
| 267 | 2020/2/3 | 15:21 | 2020/2/3 | 5:33 | Neusoft | 2020/2/3 | 20:07 |  |  |
| 271 | 2020/2/18 | 10:01 | 2020/2/18 |  | Philips Healthcare | 2020/2/18 | 20:48 |  |  |
| 272 | 2020/2/22 | 2:18 | 2020/2/22 | 1:08 | GE Healthcare | 2020/2/22 | 13:15 |  |  |
| 280 | 2020/3/1 | 19:46 | 2020/3/1 | 6:27 | GE Healthcare | 2020/3/16 | 18:26 |  |  |
| 284 | 2020/3/25 | 13:10 | 2020/3/25 | 8:10 | Philips Healthcare | 2020/3/25 | 12:04 |  |  |
| 293 | 2020/4/21 | 13:13 | 2020/4/21 | 11:57 | Philips Healthcare | 2020/4/21 | 23:25 |  |  |
| 299 | 2020/5/4 | 14:03 | 2020/5/4 | 13:19 | Philips Healthcare | 2020/5/18 | 13:29 |  |  |
| 305 | 2020/5/11 | 8:18 | 2020/5/11 | 16:01 | Philips Healthcare | 2020/5/28 | 23:50 |  |  |
| 308 | 2020/5/18 | 9:18 | 2020/5/18 | 8:37 | Philips Healthcare | 2020/5/30 | 3:15 |  |  |
| 313 | 2020/5/21 | 15:13 | 2020/5/21 | 14:41 | Philips Healthcare | 2020/6/10 | 20:16 |  |  |
| 327 | 2020/6/14 | 15:45 | 2020/6/14 |  | Philips Healthcare | 2020/6/25 | 20:07 |  |  |
| 350 | 2020/6/30 | 1:39 | 2020/6/30 | 1:19 | GE Healthcare | 2020/6/30 | 14:13 |  |  |
| 352 | 2020/7/13 | 8:06 | 2020/7/13 | 10:12 | GE Healthcare | 2020/7/21 | 12:26 |  |  |
| 363 | 2020/7/16 | 11:19 | 2020/7/16 | 11:51 | Philips Healthcare | 2020/7/22 | 16:42 |  |  |
| 368 | 2020/8/23 | 22:39 | 2020/8/23 | 13:39 | GE Healthcare | 2020/8/24 | 4:13 |  |  |
| 372 | 2020/10/15 | 14:15 | 2020/10/15 | 11:40 | Philips Healthcare | 2020/10/22 | 16:33 |  |  |
| 380 | 2020/10/22 | 8:39 | 2020/10/22 |  | Philips Healthcare | 2020/11/9 | 7:07 |  |  |
| 383 | 2020/11/2 | 19:49 | 2020/11/2 | 19:34 | GE Healthcare | 2020/11/2 | 21:11 |  |  |
| 386 | 2020/11/10 | 10:53 | 2020/11/10 | 11:37 | GE Healthcare | 2020/11/27 | 7:31 |  |  |
| 394 | 2020/11/10 | 0:01 | 2020/11/10 | 23:42 | GE Healthcare | 2020/11/11 | 8:09 |  |  |
| 398 | 2020/11/20 | 5:50 | 2020/11/20 | 6:11 | GE Healthcare | 2020/11/20 | 13:13 |  |  |
| 399 | 2020/11/29 | 9:32 | 2020/11/29 | 5:59 | Philips Healthcare | 2020/12/12 | 5:56 |  |  |
| 403 | 2020/12/2 | 16:13 | 2020/12/2 |  | Philips Healthcare | 2020/12/2 | 21:43 |  |  |
| 404 | 2020/12/2 | 21:51 | 2020/12/2 | 21:11 | GE Healthcare | 2020/12/21 | 9:35 |  |  |
| 406 | 2020/12/3 | 19:52 | 2020/12/3 | 19:23 | GE Healthcare | 2020/12/3 | 23:54 |  |  |
| 407 | 2020/12/6 | 13:29 | 2020/12/6 | 21:37 | GE Healthcare | 2020/12/7 | 3:04 |  |  |
| 409 | 2020/12/20 | 13:27 | 2020/12/20 | 13:02 | GE Healthcare | 2020/12/20 | 19:32 |  |  |
| 411 | 2020/12/27 | 15:58 | 2020/12/27 |  | Philips Healthcare | 2021/1/9 | 15:48 |  |  |
| 418 | 2021/1/8 | 23:37 | 2021/1/8 | 22:46 | GE Healthcare | 2021/1/9 | 4:30 |  |  |
| 419 | 2021/2/4 | 18:50 | 2021/2/4 | 5:55 | GE Healthcare | 2021/2/4 | 23:15 |  |  |
| 422 | 2021/3/23 | 10:35 | 2021/3/23 |  | GE Healthcare | 2021/4/4 | 14:24 |  |  |
| 425 | 2021/6/7 | 19:54 | 2021/6/7 | 19:22 | GE Healthcare | 2021/6/7 | 23:14 |  |  |
| 432 | 2021/6/22 | 18:25 | 2021/6/22 | 13:06 | GE Healthcare |  |  | 2021/6/22 | 21:51 |
| 434 | 2021/7/26 | 20:31 | 2021/7/26 | 20:25 | GE Healthcare | 2021/7/26 | 23:05 |  |  |
| 442 | 2021/9/6 | 17:56 | 2021/9/6 | 17:14 | GE Healthcare | 2021/9/14 | 19:08 |  |  |
| 449 | 2021/9/10 | 5:59 | 2021/9/10 | 5:50 | GE Healthcare | 2021/9/10 | 9:47 |  |  |
| 455 | 2021/9/11 | 12:21 | 2021/9/11 |  | Philips Healthcare | 2021/9/29 | 2:21 |  |  |
| 460 | 2021/9/25 | 11:44 | 2021/9/25 | 11:06 | Philips Healthcare | 2021/10/9 | 16:16 |  |  |
| 465 | 2021/10/3 | 16:02 | 2021/10/3 | 15:19 | Philips Healthcare | 2021/10/3 | 18:50 |  |  |
| 468 | 2021/11/15 | 15:49 | 2021/11/15 | 8:42 | Philips Healthcare | 2021/11/23 | 1:03 |  |  |
| 473 | 2021/11/16 | 13:15 | 2021/11/16 | 12:23 | GE Healthcare | 2021/11/16 | 19:39 |  |  |
| 475 | 2021/12/17 | 21:06 | 2021/12/17 | 20:42 | GE Healthcare | 2021/12/25 | 11:31 |  |  |
| 479 | 2022/1/22 | 12:31 | 2022/1/22 |  | Philips Healthcare | 2022/2/6 | 6:50 |  |  |
| 480 | 2022/2/3 | 1:32 | 2022/2/3 | 0:35 | GE Healthcare | 2022/2/3 | 12:50 |  |  |
| 486 | 2022/2/11 | 15:55 | 2022/2/11 | 5:23 | Philips Healthcare | 2022/2/27 | 8:49 |  |  |
| 496 | 2022/2/20 | 9:28 | 2022/2/20 |  | GE Healthcare | 2022/3/8 | 19:41 |  |  |
| 511 | 2022/2/25 | 1:14 | 2022/2/25 | 23:59 | GE Healthcare | 2022/2/26 | 11:13 |  |  |
| 524 | 2022/3/2 | 14:05 | 2022/3/2 | 12:56 | GE Healthcare | 2022/3/2 | 18:29 |  |  |
| 525 | 2022/3/8 | 8:19 | 2022/3/8 | 7:05 | Canon Medical Systems | 2022/3/8 | 17:53 |  |  |
| 528 | 2022/3/17 | 1:40 | 2022/3/17 | 0:36 | GE Healthcare | 2022/3/17 | 21:07 |  |  |
| 530 | 2022/4/5 | 17:02 | 2022/4/5 |  | Canon Medical Systems | 2022/4/16 | 4:23 |  |  |
| 535 | 2022/4/27 | 13:27 | 2022/4/27 | 12:46 | Philips Healthcare | 2022/5/9 | 9:13 |  |  |
| 540 | 2022/5/12 | 6:23 | 2022/5/12 | 14:17 | GE Healthcare | 2022/5/17 | 20:45 |  |  |
| 543 | 2022/6/14 | 14:21 | 2022/6/14 | 18:15 | Philips Healthcare | 2022/6/14 | 21:51 |  |  |
| 551 | 2022/6/25 | 11:23 | 2022/6/25 | 11:01 | GE Healthcare | 2022/6/25 | 22:15 |  |  |
| 557 | 2022/8/18 | 10:30 | 2022/8/18 |  | GE Healthcare | 2022/8/31 | 9:17 |  |  |
| 570 | 2022/8/24 | 16:07 | 2022/8/24 | 15:21 | Philips Healthcare | 2022/8/24 | 21:58 |  |  |
| 580 | 2022/9/9 | 14:17 | 2022/9/9 | 11:31 | Philips Healthcare | 2022/9/26 | 7:46 |  |  |
| 583 | 2022/9/14 | 15:29 | 2022/9/14 | 14:37 | Philips Healthcare | 2022/9/14 | 10:13 |  |  |
| 584 | 2022/9/15 | 18:32 | 2022/9/15 | 5:36 | GE Healthcare | 2022/9/15 | 10:17 |  |  |
| 589 | 2022/10/10 | 20:23 | 2022/10/10 | 19:54 | GE Healthcare | 2022/10/23 | 1:16 |  |  |
| 595 | 2022/11/8 | 18:42 | 2022/11/8 | 18:08 | GE Healthcare | 2022/11/9 | 9:37 |  |  |
| 606 | 2022/11/20 | 16:11 | 2022/11/20 |  | Philips Healthcare | 2022/12/3 | 13:01 |  |  |
| 611 | 2022/11/28 | 13:00 | 2022/11/28 | 12:41 | GE Healthcare | 2022/11/28 | 16:19 |  |  |
| 612 | 2022/12/6 | 15:12 | 2022/12/6 | 14:36 | Canon Medical Systems | 2022/12/7 | 2:35 |  |  |
| 620 | 2023/2/6 | 10:07 | 2023/2/6 | 9:41 | Philips Healthcare | 2023/2/6 | 13:42 |  |  |
| 632 | 2023/2/14 | 1:0 | 2023/2/14 | 0:43 | GE Healthcare | 2023/2/14 | 18:27 |  |  |
| 638 | 2023/3/22 | 4:55 | 2023/3/22 | 4:41 | GE Healthcare | 2023/4/8 | 15:56 |  |  |
| 645 | 2023/4/22 | 3:18 | 2023/4/22 | 2:59 | GE Healthcare | 2023/4/22 | 6:19 |  |  |
| 661 | 2023/4/23 | 14:42 | 2023/4/23 | 14:53 | GE Healthcare | 2023/5/12 | 17:09 |  |  |
| 669 | 2023/5/23 | 10:46 | 2023/5/23 | 9:59 | GE Healthcare | 2023/5/23 | 17:22 |  |  |
| 675 | 2023/7/4 | 10:17 | 2023/7/4 | 10:01 | Canon Medical Systems | 2023/7/4 | 18:33 |  |  |
| 681 | 2023/11/28 | 0:15 | 2023/11/28 | 0:01 | GE Healthcare | 2023/11/28 | 6:04 |  |  |
| 689 | 2024/1/4 | 19:36 | 2024/1/4 | 19:20 | GE Healthcare | 2024/1/14 | 6:53 |  |  |
| 696 | 2024/2/8 | 12:26 | 2024/2/8 |  | GE Healthcare | 2024/2/14 | 19:02 |  |  |
| 698 | 2024/2/21 | 23:04 | 2024/2/21 | 22:37 | GE Healthcare | 2024/2/22 | 6:53 |  |  |
| 707 | 2024/2/22 | 7:01 | 2024/2/22 | 6:11 | GE Healthcare | 2024/3/3 | 13:06 |  |  |
| 708 | 2024/2/29 | 15:42 | 2024/2/29 |  | GE Healthcare | 2024/3/12 | 7:29 |  |  |
| 711 | 2024/3/12 | 16:17 | 2024/3/12 | 15:30 | GE Healthcare | 2024/3/12 | 19:40 |  |  |
| 717 | 2024/3/28 | 9:24 | 2024/3/28 | 9:01 | Philips Healthcare | 2024/4/6 | 19:32 |  |  |
| 727 | 2024/4/2 | 14:40 | 2024/4/2 | 14:01 | GE Healthcare | 2024/4/19 | 6:20 |  |  |
| 740 | 2024/4/3 | 15:25 | 2024/4/3 |  | GE Healthcare | 2024/4/3 | 19:28 |  |  |
| 750 | 2024/4/19 | 16:11 | 2024/4/19 |  | GE Healthcare | 2024/4/24 | 13:20 |  |  |
| 755 | 2024/5/1 | 12:07 | 2024/5/1 |  | GE Healthcare | 2024/5/1 | 15:44 |  |  |
| 760 | 2024/5/5 | 11:49 | 2024/5/5 |  | Philips Healthcare | 2024/5/12 | 12:43 |  |  |
| 771 | 2024/5/29 | 11:09 | 2024/5/29 |  | GE Healthcare | 2024/6/18 | 10:00 | 2024/6/31 |  |
| 783 | 2025/4/8 | 19:13 | 2025/4/8 | 18:49 | GE Healthcare | 2025/4/9 | 5:05 |  |  |
| 797 | 2025/4/16 | 21:47 | 2025/4/16 | 21:35 | GE Healthcare | 2025/4/17 | 6:53 |  |  |
| 804 | 2021/6/1 | 8:09 | 2021/6/1 | 8:40 | Canon Medical Systems | 2021/6/20 | 14:38 |  |  |
| 815 | 2023/10/14 | 18:53 | 2023/10/14 | 18:11 | GE Healthcare | 2023/10/15 | 2:40 |  |  |
| 829 | 2023/8/30 | 12:49 | 2023/8/30 |  | GE Healthcare | 2023/8/30 | 17:42 |  |  |
| 840 | 2023/9/08 | 11:17 | 2023/9/08 | 18:21 | GE Healthcare | 2023/9/9 | 1:02 |  |  |
| 847 | 2023/9/24 | 12:58 | 2023/9/24 |  | Philips Healthcare | 2023/10/7 | 10:31 |  |  |
| 852 | 2024/1/04 | 15:30 | 2024/1/04 |  | GE Healthcare | 2024/1/19 | 12:43 |  |  |
| 861 | 2024/10/18 | 17:43 | 2024/10/18 | 16:54 | GE Healthcare | 2024/10/18 | 7:10 |  |  |
| 873 | 2024/10/25 | 14:51 | 2024/10/25 | 5:42 | GE Healthcare | 2024/11/8 | 16:48 |  |  |
| 889 | 2024/10/29 | 18:04 | 2024/10/29 |  | GE Healthcare | 2024/10/30 | 13:35 |  |  |
| 897 | 2024/11/12 | 14:12 | 2024/11/12 |  | GE Healthcare | 2024/11/18 | 6:46 |  |  |
| 901 | 2024/12/10 | 13:28 | 2024/12/10 |  | GE Healthcare | 2024/12/21 | 9:30 |  |  |
| 910 | 2024/3/22 | 0:28 | 2024/3/21 | 23:46 | GE Healthcare | 2024/3/22 | 17:27 |  |  |
| 923 | 2024/7/05 | 15:23 | 2024/7/05 | 14:25 | Philips Healthcare | 2024/7/21 | 20:17 |  |  |
| 937 | 2025/2/10 | 18:09 | 2025/2/10 | 17:58 | GE Healthcare | 2025/2/11 | 2:20 |  |  |
| 954 | 2025/3/10 | 9:46 | 2025/3/10 | 9:04 | Canon Medical Systems | 2025/3/10 | 15:04 |  |  |
| 966 | 2025/3/14 | 12:54 | 2025/3/14 | 12:10 | GE Healthcare | 2025/3/25 | 5:11 |  |  |
| 972 | 2025/5/20 | 19:08 | 2025/5/20 | 19:15 | GE Healthcare | 2025/5/21 | 4:48 |  |  |

Table S2. Key objective timestamps and initial clinical characteristics for the internal temporal test set.

| Patient_id | CTA Date | Scan Time | Blood Draw Date | Phlebotomy Time | Imaging Device | Intervention Date | Intervention Time | Death Date | Death Time |
| --- | --- | --- | --- | --- | --- | --- | --- | --- | --- |
| 974 | 2024/7/6 | 18:23 | 2024/7/6 | 16:23 | Philips Healthcare | 2024/7/31 | 9:13 |  |  |
| 977 | 2024/7/10 | 2:11 | 2024/7/10 | 3:18 | Philips Healthcare | 2024/7/10 | 13:40 |  |  |
| 979 | 2024/7/16 | 0:02 | 2024/7/16 | 23:12 | Philips Healthcare | 2024/7/23 | 10:14 |  |  |
| 985 | 2024/8/12 | 2:04 | 2024/9/15 |  | Neusoft | 2024/9/2 | 6:19 |  |  |
| 985 | 2024/9/10 | 4:25 | 2024/9/10 | 5:30 | GE Healthcare | 2024/9/10 | 20:29 |  |  |
| 990 | 2024/9/15 | 12:06 | 2024/9/15 | 13:42 | Neusoft | 2024/9/16 | 3:36 |  |  |
| 992 | 2024/9/15 | 20:09 | 2024/10/22 |  | Philips Healthcare | 2024/9/30 | 15:55 |  |  |
| 996 | 2024/9/21 | 2:11 | 2024/9/21 | 4:11 | Philips Healthcare | 2024/10/4 | 22:08 |  |  |
| 1001 | 2024/9/25 | 11:35 | 2024/9/25 | 11:26 | GE Healthcare | 2024/9/26 | 3:22 |  |  |
| 1012 | 2024/10/10 | 0:14 | 2025/1/22 |  | GE Healthcare | 2024/11/1 | 15:12 |  |  |
| 1020 | 2024/10/16 | 13:03 | 2025/4/5 |  | Neusoft | 2024/11/8 | 10:25 |  |  |
| 1026 | 2024/10/21 | 21:48 | 2024/10/21 | 22:21 | GE Healthcare | 2024/10/30 | 10:49 |  |  |
| 1027 | 2024/10/22 | 5:18 | 2024/10/22 | 4:03 | Neusoft | 2024/10/22 | 16:58 |  |  |
| 1030 | 2024/10/22 | 11:24 | 2024/10/22 | 11:30 | Neusoft | 2024/10/23 | 3:08 |  |  |
| 1032 | 2024/10/22 | 15:47 | 2024/10/22 | 16:24 | GE Healthcare | 2024/11/14 | 7:10 |  |  |
| 1034 | 2024/10/23 | 2:57 | 2024/10/23 | 4:30 | Philips Healthcare | 2024/11/1 | 9:05 |  |  |
| 1049 | 2024/12/2 | 4:38 | 2024/12/2 | 3:31 | GE Healthcare |  |  | 2024/12/2 | 9:50 |
| 1053 | 2024/12/6 | 22:58 | 2024/12/6 | 23:51 | Philips Healthcare | 2024/12/25 | 14:52 |  |  |
| 1065 | 2025/1/4 | 5:42 |  |  | GE Healthcare | 2025/1/4 | 8:32 |  |  |
| 1066 | 2025/1/4 | 22:55 | 2025/1/4 | 0:25 | Canon Medical Systems | 2025/1/17 | 14:40 |  |  |
| 1071 | 2025/1/15 | 17:58 | 2025/1/15 | 16:52 | Canon Medical Systems | 2025/2/11 | 21:15 |  |  |
| 1075 | 2025/1/22 | 19:46 | 2025/1/22 | 19:49 | Canon Medical Systems | 2025/1/22 | 1:27 |  |  |
| 1077 | 2025/2/25 | 10:39 | 2025/2/25 | 9:38 | GE Healthcare | 2025/3/21 | 21:29 |  |  |
| 1083 | 2025/3/10 | 1:03 |  |  | Philips Healthcare | 2025/3/19 | 15:39 |  |  |
| 1086 | 2025/3/22 | 19:46 |  |  | GE Healthcare | 2025/3/28 | 14:05 |  |  |
| 1100 | 2025/4/3 | 3:50 | 2025/4/3 | 2:50 | GE Healthcare | 2025/4/30 | 8:11 |  |  |
| 1102 | 2025/4/5 | 9:43 |  |  | GE Healthcare | 2025/4/5 | 19:40 |  |  |
| 1109 | 2025/4/30 | 5:51 |  |  | Philips Healthcare | 2025/5/8 | 9:27 |  |  |
| 1111 | 2025/5/4 | 19:25 | 2025/5/4 | 17:46 | GE Healthcare | 2025/5/4 | 21:02 |  |  |
| 1116 | 2025/5/20 | 2:22 | 2025/5/20 | 1:00 | GE Healthcare | 2025/5/29 | 6:32 |  |  |
| 1118 | 2025/5/25 | 7:59 | 2025/5/25 | 8:32 | GE Healthcare | 2025/5/25 | 13:18 |  |  |
| 1122 | 2025/6/8 | 17:45 |  |  | GE Healthcare | 2025/6/8 | 21:59 |  |  |
| 1113 | 2025/5/10 | 17:15 |  |  | GE Healthcare | 2025/5/10 | 7:19:00 |  |  |

Table S3. Variables and proportions involved in the missing value imputation process.

| Characteristic | Overall | test | train | val |
| --- | --- | --- | --- | --- |
|  | N = 150 | N = 30 | N = 90 | N = 30 |
| BMI | 53(35.3%) | 10(33.3%) | 32(35.6%) | 11(36.7%) |
| SBP, mmHg | 62(41.3%) | 10(33.3%) | 38(42.2%) | 11(36.7%) |
| DBP, mmHg | 62(41.3%) | 10(33.3%) | 38(42.2%) | 11(36.7%) |
| Laboratory examination |  |  |  |  |
| Hemoglobin, g/L | 40(26.7%) | 11(36.7%) | 20(22.2%) | 9(30.0%) |
| WBC,109/L | 40(26.7%) | 11(36.7%) | 20(22.2%) | 9(30.0%) |
| Platelet,109/L | 40(26.7%) | 11(36.7%) | 20(22.2%) | 9(30.0%) |
| CRP, mg/L | 42(28.0%) | 11(36.7%) | 22(24.4%) | 9(30.0%) |
| Fibrinogen, g/L | 41(27.3%) | 12(40.0%) | 20(22.2%) | 9(30.0%) |
| D-Dimer, mg/dL | 42(28.0%) | 11(36.7%) | 22(24.4%) | 9(30.0%) |
| RBG, mmol/L | 33(22.0%) | 8(26.7%) | 18(20.0%) | 7(23.3%) |
| TC, mmol/L | 41(27.3%) | 7(23.3%) | 26(28.9%) | 8(26.7%) |
| Triacylglycerol, mmol/L | 41(27.3%) | 7(23.3%) | 26(28.9%) | 8(26.7%) |
| HDL-C, mmol/L | 41(27.3%) | 7(23.3%) | 26(28.9%) | 8(26.7%) |
| LDL-C, mmol/L | 41(27.3%) | 7(23.3%) | 26(28.9%) | 8(26.7%) |
| Creatinine, mmol/L | 44(29.3%) | 9(30.0%) | 21(23.3%) | 10(33.3%) |

BMI: body mass index; SBP: systolic blood pressure; DBP: diastolic blood pressure; WBC: White blood cell; CRP: C-reactive protein; RBG: Random blood glucose; TC: Total cholesterol; HDL-C: High-density lipoprotein cholesterol; LDL-C: Low-density lipoprotein cholesterol.Table S4. Data augmentation strategies and parameters.

| Augmentation | Parameters | Probability | Medical Rationale |
| --- | --- | --- | --- |
| Random horizontal flip | - | 0.5 | Anatomical symmetry |
| Random vertical flip | - | 0.5 | Viewpoint invariance |
| Random rotation | ±30° | 1 | Patient positioning variation |
| Random scale | 0.6-1.0 | 1 | Field-of-view differences |
| Color jitter | Brightness=0.4, Contrast=0.4 | 1 | Scanner calibration differences |
| Gaussian blur | σ=0.1-2.0 | 0.3 | Reconstruction kernel variation |
| Random affine | Translation=15% | 1 | Slight patient movement |
| Random erasing | Scale=(0.02, 0.15) | 0.3 | Simulates artifacts or occlusions |

Table S5. Time required for volume-of-interest initialization by three radiologists.

| Patient_id | Time A (s) | Time B (s) | Time C (s) |
| --- | --- | --- | --- |
| 13 | 52 | 46 | 33 |
| 32 | 53 | 42 | 49 |
| 175 | 39 | 51 | 59 |
| 179 | 36 | 33 | 31 |
| 273 | 55 | 43 | 45 |
| 372 | 44 | 45 | 44 |
| 383 | 42 | 54 | 36 |
| 423 | 48 | 49 | 39 |
| 449 | 41 | 47 | 55 |
| 457 | 43 | 35 | 46 |
| 483 | 34 | 52 | 57 |
| 511 | 57 | 39 | 42 |
| 555 | 32 | 58 | 40 |
| 611 | 37 | 41 | 51 |
| 645 | 38 | 40 | 38 |
| 697 | 41 | 37 | 53 |
| 720 | 56 | 56 | 37 |
| 732 | 49 | 50 | 41 |
| 955 | 45 | 36 | 47 |
| 972 | 51 | 44 | 34 |

Table S6. Inter-operator variability in the total slice count.

| Comparison Between Annotators | Median Absolute Difference | Interquartile Range |
| --- | --- | --- |
| A vs. B | 2 slices | 1–3 slices |
| A vs. C | 2 slices | 1–3 slices |
| B vs. C | 2 slices | 1–4 slices |

Table S7. Technical specifications of the evaluated backbone networks.

| Backbone | Parameters | Pretraining | Feature Dimension |
| --- | --- | --- | --- |
| ResNet-18 | 11.7M | ImageNet-1k | 512 |
| ResNet-50 | 25.6M | ImageNet-1k | 2,048 |
| ResNet-101 | 44.5M | ImageNet-1k | 2,048 |
| DenseNet-121 | 8.0M | ImageNet-1k | 1,024 |
| VGG-16 | 138M | ImageNet-1k | 25,088 |
| Vision Transformer-B/16 | 86M | ImageNet-21k → ImageNet-1k | 768 |
| AlexNet | 61.1M | ImageNet-1k | 4,096 |
| MedViT-Large | 183M | ImageNet-1k | 1,000 |

Table S8. Model training hyperparameters.

| Hyperparameter | Value | Rationale |
| --- | --- | --- |
| Optimizer | AdamW | Combines Adam advantages with decoupled weight decay |
| Base learning rate | 3×10⁻^5^ | Stable convergence for medical imaging tasks |
| Weight decay | 1×10⁻⁵ | Moderate regularization to prevent overfitting |
| Beta coefficients | (0.9, 0.999) | Standard momentum settings |
| Learning rate scheduler | OneCycleLR | Fast convergence with automatic warm-up and annealing |
| Maximum learning rate | 6×10⁻⁴ | 3× base rate as recommended for OneCycleLR |
| Batch size | 1 (effective: 4) | GPU memory constraints with 3D volumes |
| Gradient accumulation steps | 4 | Achieves effective batch size of 4 |
| Maximum epochs | 200 | Sufficient for convergence with early stopping |
| Early stopping patience | 30 epochs | Based on validation AUC plateau |
| Mixed precision training | Enabled (AMP) | 2-3× training acceleration with minimal accuracy loss |

Table S9. Comparison of additional baseline characteristics between the stable group and the impending rupture group.

| Characteristic | Overall | Stable | Impending rupture | Statistic^1^ | p-value^1^ |
| --- | --- | --- | --- | --- | --- |
|  | N = 150 | N = 75 | N = 75 |  |  |
| BMI | 23.4 (21.8, 26.3) | 23.6 (21.6, 26.7) | 23.4 (22.0, 24.0) | 146.5 | 0.572 |
| Arrhythmia, n (%) | 6 (4.0%) | 3 (4.0%) | 3 (4.0%) |  | >0.999 |
| CHD, n (%) | 25 (16.7%) | 15 (20.0%) | 10 (13.3%) | 1.2 | 0.273 |
| Prior MI, n (%) | 12 (8.0%) | 6 (8.0%) | 6 (8.0%) | 0 | >0.999 |
| Prior Stroke, n (%) | 17 (11.3%) | 10 (13.3%) | 7 (9.3%) | 0.6 | 0.44 |
| Haemorrhagic, n (%) | 3 (2.0%) | 3 (4.0%) | 0 (0.0%) |  | 0.245 |
| Ischaemic, n (%) | 14 (9.3%) | 7 (9.3%) | 7 (9.3%) | 0 | >0.999 |
| Laboratory examination |  |  |  |  |  |
| HDL-C, mmol/L | 0.99 (0.88, 1.17) | 0.98 (0.86, 1.10) | 1.00 (0.88, 1.18) | 174.5 | 0.844 |
| Maximum AAA diameter, mm | 49 (41, 61) | 49 (41, 61) | 50 (40, 61) | 2,906.50 | 0.725 |
| Prior medications |  |  |  |  |  |
| OHD, n (%) | 5 (3.3%) | 4 (5.3%) | 1 (1.3%) |  | 0.367 |
| RASS inhibitors, n (%) | 2 (1.3%) | 2 (2.7%) | 0 (0.0%) |  | 0.497 |
| Β-blockers, n (%) | 1 (0.7%) | 1 (1.3%) | 0 (0.0%) |  | >0.999 |
| CCB, n (%) | 5 (3.3%) | 5 (6.7%) | 0 (0.0%) |  | 0.058 |

Table S10. Comparison of baseline characteristics among training, validation, and test sets.

| Characteristic | Overall | test | train | val |
| --- | --- | --- | --- | --- |
|  | N = 150 | N = 30 | N = 90 | N = 30 |
| Age | 69 (64, 73) | 69 (61, 73) | 69 (65, 74) | 68 (63, 73) |
| Male, n (%) | 134 (89.3%) | 27 (90.0%) | 80 (88.9%) | 27 (90.0%) |
| BMI | 23.4 (21.8, 26.3) | 23.3 (22.2, 30.0) | 23.6 (22.0, 26.4) | 23.5 (20.2, 26.2) |
| Smoking, n (%) | 30 (20.0%) | 5 (16.7%) | 18 (20.0%) | 7 (23.3%) |
| Drinking, n (%) | 19 (12.7%) | 4 (13.3%) | 12 (13.3%) | 3 (10.0%) |
| Hypertension, n (%) | 42 (28.0%) | 7 (23.3%) | 26 (28.9%) | 9 (30.0%) |
| Diabetes, n (%) | 10 (6.7%) | 0 (0.0%) | 9 (10.0%) | 1 (3.3%) |
| Arrhythmia, n (%) | 6 (4.0%) | 0 (0.0%) | 4 (4.4%) | 2 (6.7%) |
| CHD, n (%) | 25 (16.7%) | 4 (13.3%) | 15 (16.7%) | 6 (20.0%) |
| Prior MI, n (%) | 12 (8.0%) | 3 (10.0%) | 6 (6.7%) | 3 (10.0%) |
| Prior Stroke, n (%) | 17 (11.3%) | 2 (6.7%) | 12 (13.3%) | 3 (10.0%) |
| Haemorrhagic, n (%) | 3 (2.0%) | 1 (3.3%) | 2 (2.2%) | 0 (0.0%) |
| Ischaemic, n (%) | 14 (9.3%) | 1 (3.3%) | 10 (11.1%) | 3 (10.0%) |
| Prior surgery, n (%) | 18 (12.0%) | 2 (6.7%) | 11 (12.2%) | 5 (16.7%) |
| SBP, mmHg | 136 (120, 148) | 140 (125, 156) | 136 (116, 152) | 134 (119, 143) |
| DBP, mmHg | 78 (72, 94) | 83 (76, 98) | 78 (70, 94) | 78 (71, 89) |
| Laboratory examination |  |  |  |  |
| Hemoglobin, g/L | 116 (102, 136) | 118 (94, 143) | 116 (101, 135) | 116 (103, 139) |
| WBC,10^9^/L | 9.7 (7.1, 13.9) | 10.9 (7.7, 13.4) | 9.4 (6.3, 13.8) | 10.1 (7.8, 14.9) |
| Platelet,10^9^/L | 187 (159, 222) | 184 (144, 222) | 186 (159, 223) | 199 (164, 213) |
| CRP, mg/L | 12 (6, 44) | 44 (9, 55) | 11 (5, 38) | 10 (4, 28) |
| Fibrinogen, g/L | 3.31 (2.73, 4.39) | 3.74 (2.97, 5.04) | 3.30 (2.66, 4.31) | 3.19 (2.80, 4.21) |
| D-Dimer, mg/dL | 5 (2, 12) | 3 (1, 7) | 6 (3, 14) | 4 (3, 11) |
| FBG, mmol/L | 7.0 (5.6, 9.9) | 7.9 (5.7, 11.0) | 6.8 (5.5, 8.8) | 7.4 (6.0, 17.0) |
| TC, mmol/L | 4.27 (3.72, 5.00) | 4.08 (3.07, 5.01) | 4.33 (3.80, 4.62) | 4.23 (3.35, 5.94) |
| Triacylglycerol, mmol/L | 1.26 (1.00, 2.04) | 2.04 (0.78, 2.55) | 1.23 (1.03, 1.79) | 1.38 (0.99, 2.13) |
| HDL-C, mmol/L | 0.99 (0.88, 1.17) | 1.00 (0.70, 1.24) | 0.98 (0.88, 1.02) | 1.11 (0.89, 1.43) |
| LDL-C, mmol/L | 2.52 (2.17, 2.98) | 2.72 (1.67, 2.99) | 2.54 (2.17, 2.90) | 2.38 (2.17, 3.35) |
| Creatinine, mmol/L | 89 (66, 136) | 79 (65, 157) | 100 (72, 134) | 86 (78, 138) |
| MAX Diameter, mm | 49 (41, 61) | 46 (34, 61) | 50 (43, 60) | 48 (37, 61) |
| Prior medications |  |  |  |  |
| OHD, n (%) | 5 (3.3%) | 0 (0.0%) | 5 (5.6%) | 0 (0.0%) |
| RASS inhibitors, n (%) | 2 (1.3%) | 0 (0.0%) | 2 (2.2%) | 0 (0.0%) |
| Statins, n (%) | 21 (14.0%) | 3 (10.0%) | 15 (16.7%) | 3 (10.0%) |
| Β-blockers, n (%) | 1 (0.7%) | 0 (0.0%) | 1 (1.1%) | 0 (0.0%) |
| Aspirin, n (%) | 24 (16.0%) | 2 (6.7%) | 16 (17.8%) | 6 (20.0%) |
| CCB, n (%) | 5 (3.3%) | 0 (0.0%) | 4 (4.4%) | 1 (3.3%) |

Categorical variables are reported as frequency and percentage (n, %).

Continuous variables are reported as median and interquartile range med(Q1-Q3).

BMI: Body mass index; CHD: Coronary atherosclerotic heart disease; MI: Myocardial infarction; SBP: systolic blood pressure; DBP: diastolic blood pressure; WBC: White blood cell; CRP: C-reactive protein; RBG: Random blood glucose; TC: Total cholesterol; HDL-C: High-density lipoprotein cholesterol; LDL-C: Low-density lipoprotein cholesterol; OHD: Oral hypoglycemic drugs; RASS: Renin-angiotensin-aldosterone system; CCB: Calcium channel blockers.

Table S11.Comparison of baseline characteristics between the stable and impending rupture groups after KNN imputation.

| Characteristic | Overall | Stable | Impending rupture | Statistic^1^ | p-value^1^ |
| --- | --- | --- | --- | --- | --- |
|  | N = 150 | N = 75 | N = 75 |  |  |
| Age | 69 (64, 73) | 68 (63, 73) | 70 (65, 74) | 2,502.00 | 0.243 |
| Male, n (%) | 134 (89.3%) | 68 (90.7%) | 66 (88.0%) | 0.28 | 0.597 |
| BMI | 23.88 (23.13, 24.72) | 23.55 (22.74, 24.72) | 24.06 (23.32, 24.75) | 2,449.00 | 0.172 |
| Smoking, n (%) | 30 (20.0%) | 21 (28.0%) | 9 (12.0%) | 6 | 0.014 |
| Drinking, n (%) | 19 (12.7%) | 14 (18.7%) | 5 (6.7%) | 4.88 | 0.027 |
| Hypertension, n (%) | 42 (28.0%) | 27 (36.0%) | 15 (20.0%) | 4.76 | 0.029 |
| Diabetes, n (%) | 10 (6.7%) | 8 (10.7%) | 2 (2.7%) | 3.86 | 0.05 |
| Arrhythmia, n (%) | 6 (4.0%) | 3 (4.0%) | 3 (4.0%) |  | >0.999 |
| CHD, n (%) | 25 (16.7%) | 15 (20.0%) | 10 (13.3%) | 1.2 | 0.273 |
| Prior MI, n (%) | 12 (8.0%) | 6 (8.0%) | 6 (8.0%) | 0 | >0.999 |
| Prior Stroke, n (%) | 17 (11.3%) | 10 (13.3%) | 7 (9.3%) | 0.6 | 0.44 |
| Haemorrhagic, n (%) | 3 (2.0%) | 3 (4.0%) | 0 (0.0%) |  | 0.245 |
| Ischaemic, n (%) | 14 (9.3%) | 7 (9.3%) | 7 (9.3%) | 0 | >0.999 |
| Prior surgery, n (%) | 18 (12.0%) | 13 (17.3%) | 5 (6.7%) | 4.04 | 0.044 |
| SBP, mmHg | 136 (125, 146) | 139 (128, 147) | 135 (117, 145) | 3,211.50 | 0.134 |
| DBP, mmHg | 82 (76, 90) | 85 (79, 93) | 78 (73, 86) | 3,935.00 | <0.001 |
| Laboratory examination |  |  |  |  |  |
| Hemoglobin, g/L | 118 (106, 131) | 123 (113, 137) | 113 (94, 127) | 3,706.50 | <0.001 |
| WBC,10^9^/L | 9.4 (7.0, 13.3) | 7.7 (6.2, 9.8) | 11.9 (8.6, 15.4) | 1,398.00 | <0.001 |
| Platelet,10^9^/L | 188 (165, 222) | 181 (165, 216) | 191 (165, 232) | 2,485.00 | 0.219 |
| CRP, mg/L | 16 (9, 38) | 16 (8, 24) | 18 (9, 46) | 2,429.50 | 0.151 |
| Fibrinogen, g/L | 3.33 (2.96, 4.39) | 3.32 (3.02, 4.31) | 3.33 (2.80, 4.51) | 2,841.00 | 0.916 |
| D-Dimer, mg/dL | 6 (3, 11) | 6 (3, 11) | 6 (3, 12) | 2,705.00 | 0.688 |
| FBG, mmol/L | 7.1 (6.3, 9.0) | 6.8 (6.2, 8.0) | 7.8 (6.4, 11.0) | 2,008.50 | 0.003 |
| TC, mmol/L | 4.24 (4.08, 4.39) | 4.28 (4.15, 4.60) | 4.19 (3.93, 4.31) | 3,703.50 | <0.001 |
| Triacylglycerol, mmol/L | 1.41 (1.20, 1.64) | 1.42 (1.20, 1.60) | 1.41 (1.19, 1.65) | 2,744.00 | 0.798 |
| HDL-C, mmol/L | 0.96 (0.91, 1.03) | 0.96 (0.91, 1.04) | 0.96 (0.90, 1.03) | 2,964.00 | 0.570 |
| LDL-C, mmol/L | 2.52 (2.41, 2.62) | 2.55 (2.45, 2.67) | 2.49 (2.37, 2.57) | 3,510.50 | 0.009 |
| Creatinine, mmol/L | 95 (74, 128) | 96 (79, 122) | 89 (72, 147) | 2,828.00 | 0.955 |
| MAX Diameter, mm | 49 (41, 61) | 49 (41, 61) | 50 (40, 61) | 2,906.50 | 0.725 |
| Prior medications |  |  |  |  |  |
| OHD, n (%) | 5 (3.3%) | 4 (5.3%) | 1 (1.3%) |  | 0.367 |
| RASS inhibitors, n (%) | 2 (1.3%) | 2 (2.7%) | 0 (0.0%) |  | 0.497 |
| Statins, n (%) | 21 (14.0%) | 13 (17.3%) | 8 (10.7%) | 1.38 | 0.239 |
| Β-blockers, n (%) | 1 (0.7%) | 1 (1.3%) | 0 (0.0%) |  | >0.999 |
| Aspirin, n (%) | 24 (16.0%) | 18 (24.0%) | 6 (8.0%) | 7.14 | 0.008 |
| CCB, n (%) | 5 (3.3%) | 5 (6.7%) | 0 (0.0%) |  | 0.058 |

Categorical variables are reported as frequency and percentage (n, %).

Continuous variables are reported as median and interquartile range med(Q1-Q3).

BMI: Body mass index; CHD: Coronary atherosclerotic heart disease; MI: Myocardial infarction; SBP: systolic blood pressure; DBP: diastolic blood pressure; WBC: White blood cell; CRP: C-reactive protein; RBG: Random blood glucose; TC: Total cholesterol; HDL-C: High-density lipoprotein cholesterol; LDL-C: Low-density lipoprotein cholesterol; OHD: Oral hypoglycemic drugs; RASS: Renin-angiotensin-aldosterone system; CCB: Calcium channel blockers.

Table S12. Comparison of baseline characteristics among training, validation, and test sets after KNN imputation.

| Characteristic | Overall | test | train | val |
| --- | --- | --- | --- | --- |
|  | N = 150 | N = 30 | N = 90 | N = 30 |
| Age | 69 (64, 73) | 69 (61, 73) | 69 (65, 74) | 68 (63, 73) |
| Male, n (%) | 134 (89.3%) | 27 (90.0%) | 80 (88.9%) | 27 (90.0%) |
| BMI | 23.88 (23.13, 24.72) | 23.65 (23.13, 24.54) | 24.07 (23.00, 24.79) | 23.53 (23.18, 24.50) |
| Smoking, n (%) | 30 (20.0%) | 5 (16.7%) | 18 (20.0%) | 7 (23.3%) |
| Drinking, n (%) | 19 (12.7%) | 4 (13.3%) | 12 (13.3%) | 3 (10.0%) |
| Hypertension, n (%) | 42 (28.0%) | 7 (23.3%) | 26 (28.9%) | 9 (30.0%) |
| Diabetes, n (%) | 10 (6.7%) | 0 (0.0%) | 9 (10.0%) | 1 (3.3%) |
| Arrhythmia, n (%) | 6 (4.0%) | 0 (0.0%) | 4 (4.4%) | 2 (6.7%) |
| CHD, n (%) | 25 (16.7%) | 4 (13.3%) | 15 (16.7%) | 6 (20.0%) |
| Prior MI, n (%) | 12 (8.0%) | 3 (10.0%) | 6 (6.7%) | 3 (10.0%) |
| Prior Stroke, n (%) | 17 (11.3%) | 2 (6.7%) | 12 (13.3%) | 3 (10.0%) |
| Haemorrhagic, n (%) | 3 (2.0%) | 1 (3.3%) | 2 (2.2%) | 0 (0.0%) |
| Ischaemic, n (%) | 14 (9.3%) | 1 (3.3%) | 10 (11.1%) | 3 (10.0%) |
| Prior surgery, n (%) | 18 (12.0%) | 2 (6.7%) | 11 (12.2%) | 5 (16.7%) |
| SBP, mmHg | 136 (125, 146) | 142 (130, 154) | 135 (120, 145) | 138 (129, 146) |
| DBP, mmHg | 136 (125, 146) | 136 (125, 146) | 81 (76, 88) | 80 (73, 90) |
| Laboratory examination |  |  |  |  |
| Hemoglobin, g/L | 118 (106, 131) | 119 (109, 125) | 117 (105, 134) | 122 (110, 128) |
| WBC,10^9^/L | 9.4 (7.0, 13.3) | 8.1 (7.1, 11.9) | 10.7 (7.2, 13.4) | 8.6 (6.5, 13.7) |
| Platelet,10^9^/L | 188 (165, 222) | 179 (171, 210) | 192 (164, 224) | 181 (168, 213) |
| CRP, mg/L | 16 (9, 38) | 23 (11, 63) | 15 (8, 25) | 17 (9, 37) |
| Fibrinogen, g/L | 3.33 (2.96, 4.39) | 3.28 (3.02, 5.01) | 3.59 (2.82, 4.31) | 3.20 (3.01, 4.21) |
| D-Dimer, mg/dL | 6 (3, 11) | 6 (2, 8) | 7 (4, 13) | 5 (3, 10) |
| RBG, mmol/L | 7.1 (6.3, 9.0) | 7.2 (6.6, 8.4) | 7.1 (5.9, 9.0) | 6.9 (6.4, 10.0) |
| TC, mmol/L | 4.24 (4.08, 4.39) | 4.27 (4.08, 4.32) | 4.22 (3.96, 4.52) | 4.25 (4.19, 4.30) |
| Triacylglycerol, mmol/L | 1.41 (1.20, 1.64) | 1.47 (1.20, 1.70) | 1.40 (1.19, 1.58) | 1.45 (1.20, 1.61) |
| HDL-C, mmol/L | 0.96 (0.91, 1.03) | 0.97 (0.95, 1.14) | 0.95 (0.89, 1.00) | 1.01 (0.95, 1.12) |
| LDL-C, mmol/L | 2.52 (2.41, 2.62) | 2.53 (2.48, 2.57) | 2.51 (2.38, 2.62) | 2.52 (2.48, 2.62) |
| Creatinine, mmol/L | 95 (74, 128) | 83 (66, 127) | 101 (76, 131) | 86 (75, 122) |
| MAX Diameter, mm | 49 (41, 61) | 46 (34, 61) | 50 (43, 60) | 48 (37, 61) |
| Prior medications |  |  |  |  |
| OHD, n (%) | 5 (3.3%) | 0 (0.0%) | 5 (5.6%) | 0 (0.0%) |
| RASS inhabitors, n (%) | 2 (1.3%) | 0 (0.0%) | 2 (2.2%) | 0 (0.0%) |
| Statins, n (%) | 21 (14.0%) | 3 (10.0%) | 15 (16.7%) | 3 (10.0%) |
| Β-blockers, n (%) | 1 (0.7%) | 0 (0.0%) | 1 (1.1%) | 0 (0.0%) |
| Aspirin, n (%) | 24 (16.0%) | 2 (6.7%) | 16 (17.8%) | 6 (20.0%) |
| CCB, n (%) | 5 (3.3%) | 0 (0.0%) | 4 (4.4%) | 1 (3.3%) |

Categorical variables are reported as frequency and percentage (n, %).

Continuous variables are reported as median and interquartile range med(Q1-Q3).

BMI: Body mass index; CHD: Coronary atherosclerotic heart disease; MI: Myocardial infarction; SBP: systolic blood pressure; DBP: diastolic blood pressure; WBC: White blood cell; CRP: C-reactive protein; RBG: Random blood glucose; TC: Total cholesterol; HDL-C: High-density lipoprotein cholesterol; LDL-C: Low-density lipoprotein cholesterol; OHD: Oral hypoglycemic drugs; RASS: Renin-angiotensin-aldosterone system; CCB: Calcium channel blockers.

Table S13. Bootstrap stability analysis and composite evaluation of feature selection methods.

| Feature selection method | AUC | Bootstrap stability | Composite score | Number of features |
| --- | --- | --- | --- | --- |
| GBDT | 0.680 | 28.45% | 0.522 | 4 |
| GBDT | 0.827 | 36.85% | 0.644 | 6 |
| GBDT | 0.862 | 35.88% | 0.661 | 5 |
| Logistic Regression | 0.853 | 40.10% | 0.672 | 4 |
| Logistic Regression | 0.880 | 44.24% | 0.705 | 5 |
| Logistic Regression | 0.738 | 46.52% | 0.629 | 6 |
| mRMR | 0.551 | 19.92% | 0.410 | 6 |
| mRMR | 0.773 | 20.98% | 0.548 | 5 |
| mRMR | 0.813 | 17.45% | 0.558 | 4 |
| Random Forest | 0.836 | 50.86% | 0.705 | 5 |
| **Random Forest** | **0.836** | **55.55%** | **0.724** | **6** |
| Random Forest | 0.876 | 45.35% | 0.707 | 4 |
| XGBoost | 0.542 | 13.60% | 0.380 | 4 |
| XGBoost | 0.764 | 17.28% | 0.528 | 5 |
| XGBoost | 0.747 | 23.77% | 0.543 | 6 |

The composite score is calculated as (0.6 * AUC) + (0.4 * Stability). The model with the highest composite score is highlighted in bold. AUC: Area under the curve.

Table S14. Pairwise comparison of AUCs using the DeLong test.

| Comparison | Model 1 AUC (95% CI) | Model 2 AUC (95% CI) | AUC Difference | DeLong P-value |
| --- | --- | --- | --- | --- |
| Deep Learning vs. Simple Clinical Model | 0.898 (0.737–1.000) | 0.751 (0.560–0.903) | 0.147 | 0.838 |
| Deep Learning vs. CTA Features Model | 0.898 (0.737–1.000) | 0.778 (0.607–0.928) | 0.12 | 0.899 |

AUC: Area under the curve; CTA: Computed tomography angiography.

Table S15. Detailed per-patient runtime breakdown of the proposed model on the internal temporal test set.

| Patient_id | data_loading | core_inference_without_tta | tta_processing | grad_cam_generation | io_operations | per_patient_total |
| --- | --- | --- | --- | --- | --- | --- |
| 974 | 0.01 | 2.22 | 0.98 | 7.43 | 0.01 | 10.65 |
| 977 | 0.01 | 0.03 | 0.97 | 6.85 | 0.02 | 7.87 |
| 979 | 0.01 | 0.02 | 0.96 | 6.80 | 0.03 | 7.82 |
| 985 | 0.01 | 0.03 | 0.98 | 6.94 | 0.02 | 7.97 |
| 985 | 0.01 | 0.03 | 1.03 | 7.35 | 0.01 | 8.42 |
| 990 | 0.01 | 0.03 | 1.01 | 7.46 | 0.00 | 8.51 |
| 992 | 0.01 | 0.03 | 0.95 | 6.66 | 0.03 | 7.67 |
| 996 | 0.01 | 0.03 | 1.03 | 6.71 | 0.02 | 7.79 |
| 1001 | 0.01 | 0.02 | 1.02 | 6.70 | 0.05 | 7.79 |
| 1012 | 0.01 | 0.03 | 0.95 | 7.27 | 0.01 | 8.27 |
| 1020 | 0.01 | 0.03 | 1.00 | 7.19 | 0.02 | 8.24 |
| 1026 | 0.01 | 0.03 | 0.98 | 6.88 | 0.02 | 7.91 |
| 1027 | 0.01 | 0.03 | 1.02 | 6.45 | 0.02 | 7.52 |
| 1030 | 0.01 | 0.02 | 1.08 | 6.94 | 0.02 | 8.07 |
| 1032 | 0.01 | 0.03 | 1.08 | 7.44 | 0.01 | 8.56 |
| 1034 | 0.01 | 0.03 | 1.04 | 7.29 | 0.03 | 8.39 |
| 1049 | 0.01 | 0.03 | 1.15 | 7.06 | 0.02 | 8.26 |
| 1053 | 0.01 | 0.03 | 1.13 | 7.37 | 0.02 | 8.55 |
| 1065 | 0.01 | 0.03 | 1.15 | 7.23 | 0.01 | 8.42 |
| 1066 | 0.01 | 0.03 | 1.15 | 7.39 | 0.02 | 8.58 |
| 1071 | 0.01 | 0.03 | 1.17 | 6.86 | 0.01 | 8.08 |
| 1075 | 0.01 | 0.03 | 1.19 | 6.75 | 0.02 | 7.98 |
| 1077 | 0.01 | 0.03 | 1.20 | 7.04 | 0.01 | 8.29 |
| 1083 | 0.01 | 0.03 | 1.19 | 7.28 | 0.01 | 8.51 |
| 1086 | 0.01 | 0.04 | 1.29 | 6.85 | 0.01 | 8.19 |
| 1100 | 0.01 | 0.03 | 1.29 | 6.99 | 0.03 | 8.34 |
| 1102 | 0.01 | 0.03 | 1.12 | 7.09 | 0.02 | 8.26 |
| 1109 | 0.01 | 0.03 | 1.06 | 7.46 | 0.03 | 8.58 |
| 1111 | 0.01 | 0.03 | 1.13 | 7.13 | 0.01 | 8.30 |
| 1116 | 0.01 | 0.03 | 1.09 | 6.79 | 0.01 | 7.92 |
| 1118 | 0.01 | 0.03 | 1.09 | 6.66 | 0.02 | 7.81 |
| 1122 | 0.01 | 0.03 | 1.14 | 6.93 | 0.01 | 8.12 |
| 1113 | 0.01 | 0.03 | 1.16 | 7.00 | 0.01 | 8.20 |

All times are given in seconds (s).

Table S16. Comparison of baseline characteristics between patients with complete pre-intervention data and those requiring imputation.

| Characteristic | Overall N = 150 | Imputation‑required N = 41 | Complete‑case N = 109 | Statistic | P-value |
| --- | --- | --- | --- | --- | --- |
| Age | 69 (64, 73) | 68 (63, 71) | 69 (65, 74) | 1899.5 | 0.158 |
| Male, n (%) | 134 (89.3%) | 40 (97.6%) | 94 (86.2%) |  | 0.071 |
| BMI | 23.4 (21.8, 26.3) | 23.5 (22.5, 26.2) | 23.4 (21.6, 26.4) | 80 | 0.927 |
| Smoking, n (%) | 30 (20.0%) | 4 (9.8%) | 26 (23.9%) | χ² = 3.70 | 0.054 |
| Drinking, n (%) | 19 (12.7%) | 0 (0.0%) | 19 (17.4%) | χ² = 8.18 | 0.004 |
| Hypertension, n (%) | 42 (28.0%) | 4 (9.8%) | 38 (34.9%) | χ² = 9.32 | 0.002 |
| Diabetes, n (%) | 10 (6.7%) | 0 (0.0%) | 10 (9.2%) |  | 0.062 |
| Arrhythmia, n (%) | 6 (4.0%) | 1 (2.4%) | 5 (4.6%) |  | >0.999 |
| CHD, n (%) | 25 (16.7%) | 3 (7.3%) | 22 (20.2%) | χ² = 3.55 | 0.060 |
| Prior MI, n (%) | 12 (8.0%) | 2 (4.9%) | 10 (9.2%) |  | 0.513 |
| Prior Stroke, n (%) | 17 (11.3%) | 2 (4.9%) | 15 (13.8%) |  | 0.157 |
| Haemorrhagic, n (%) | 3 (2.0%) | 0 (0.0%) | 3 (2.8%) |  | 0.562 |
| Ischaemic, n (%) | 14 (9.3%) | 2 (4.9%) | 12 (11.0%) |  | 0.352 |
| Prior surgery, n (%) | 18 (12.0%) | 4 (9.8%) | 14 (12.8%) |  | 0.780 |
| SBP, mmHg | 136 (120, 148) | 139 (127, 144) | 135 (119, 149) | 366.5 | 0.885 |
| DBP, mmHg | 78 (72, 94) | 76 (69, 89) | 78 (73, 96) | 298 | 0.432 |
| Impending rupture | 75 (50.0%) | 12 (29.3%) | 63 (57.8%) | χ² = 9.70 | 0.002 |

Categorical variables are reported as frequency and percentage (n, %).

Continuous variables are reported as median and interquartile range med(Q1-Q3).

BMI: Body mass index; CHD: Coronary atherosclerotic heart disease; MI: Myocardial infarction; SBP: systolic blood pressure; DBP: diastolic blood pressure.
